# Temporal and Spatial Trends in Benthic Infauna and Potential Drivers, in a Highly Tidal Estuary in Atlantic Canada

**DOI:** 10.1007/s12237-023-01222-w

**Published:** 2023-06-13

**Authors:** Andrew J. Guerin, Karen A. Kidd, Marie-Josée Maltais, Angella Mercer, Heather L. Hunt

**Affiliations:** 1 Department of Biology, McMaster University, Hamilton, ON Canada; 2 School of Earth, Environment and Society, McMaster University, Hamilton, ON Canada; 3 Department of Biological Sciences, University of New Brunswick, Saint John, NB Canada

**Keywords:** Marine invertebrates, Trace metal contamination, Sediment chemistry, Multivariate analysis

## Abstract

Infaunal invertebrate communities of coastal marine sediments are often impacted by human activities, particularly in harbours and estuaries. However, while many studies have attempted to identify the key factors affecting benthic infauna, few have done so for highly energetic tidal estuaries. Samples were collected over a decade (2011–2020) from a series of reference sites in Saint John Harbour (45.25° N, 66.05° W), a highly tidal estuary in the Bay of Fundy, Canada. These data were used to examine spatial and temporal trends in infaunal invertebrate communities and sediment properties and to determine the extent to which the biological patterns were driven by measured physical and chemical variables. There were substantial differences among sites in infaunal invertebrate abundance (median ranging from 688 to 13,700 individuals per square meter), infaunal species richness (median ranging from 8 to 22), and Shannon diversity (median ranging from 1.26 to 2.34); multivariate analysis also revealed variation in species composition among sites. Sediment contaminant concentrations also varied among sites, but differences tended to be smaller (e.g. median chromium concentrations ranging from 21.6 to 27.6 mg/kg). Sample contaminant concentrations were all below probable effect levels, and almost all below threshold effect levels (Canadian interim sediment quality guidelines), but relationships with biological data were still detectable. However, physical variables (depth, sediment characteristics) were better predictors of biological variables and community composition. These results confirm the importance of physical factors in shaping infaunal communities in soft-sediment habitats in tidally influenced coastal waters.

**Supplementary Information:**

The online version contains supplementary material available at 10.1007/s12237-023-01222-w.

## Introduction

Coastal marine sediments and their associated biological communities are important targets for monitoring impacts of human activities and for improving our understanding of natural variability and the responses of species to environmental changes. Sediments absorb and integrate contaminants over time (Chapman 1989) and provide habitat and nutrients for benthic infauna at the base of many marine food webs (Bilyard 1987). Infauna often respond relatively quickly to increased contaminant levels and other habitat changes, can be sampled quantitatively, have diverse and measurable responses to environmental stressors, and provide spatial information on habitat quality and anthropogenic impacts (Pearson and Rosenberg 1978; Hartley 1982). So-called ‘sensitive’ infaunal species tend to decline in abundance or disappear in response to even relatively small increases in stressor levels (Ellis et al. 2017), while ‘opportunistic’ species are more tolerant and tend to be small, rapidly maturing, and highly fecund (many are polychaetes; Glasby et al. 2021). As stressors increase, communities can become dominated by opportunists, sometimes in very high abundances (Grassle and Grassle 1974; Ward and Hutchings 1996).

Since coastal waters can be a focus for human activities, infaunal communities are affected by a range of anthropogenic stressors, including fishing (Clarke et al. 2018); dredging and dumping of dredged material (Cooper et al. 2011; Donázar-Aramendía et al. 2018); organic enrichment due to discharge of domestic sewage and agricultural run-off (Zeldis et al. 2020); and sediment contamination with heavy metals, polycyclic aromatic hydrocarbons (PAHs), polychlorinated biphenyls (PCBs), and other organic and inorganic substances (Birch 2000; Sprovieri et al. 2007; Ozcan et al. 2013). This is especially true near ports and harbours, where intense human activity can lead to highly contaminated sediments (Martínez-Lladó et al. 2007; Gibert et al. 2009). Local anthropogenic stressors will also change over time, against a backdrop of anthropogenically-driven regional and global environmental change. In this context, monitoring of change in benthic communities requires a long-term approach, including sampling of both potentially impacted sites and, ideally, multiple sites which are more distant from potential impacts. These ‘reference sites’ must be physically similar to potentially impacted sites and should represent minimally impacted conditions for the region; few truly pristine sites are likely to exist (Borja et al. 2012). Data from such sites can be used to assess natural spatial and temporal variability and to account for larger scale environmental changes. One approach is to define ‘ranges of normal’ values for site, local, and regional levels using multiple years of data from reference sites (Arciszewski and Munkittrick 2015).

Anthropogenic stressors are not the only (or necessarily the most important) factors driving infaunal abundance and composition, particularly in dynamic coastal waters. Factors such as depth, temperature, and salinity have well-established relationships with community composition (Dauer 1993; Glockzin and Zettler 2008). The physical characteristics and organic content of sediments are also important; more coarse, sandy sediments tend to have more diverse and productive communities than finer sediments with greater organic content (Thrush et al. 2004; Cooper et al. 2011; Robertson et al. 2015). Natural ‘stressors’ which can affect infaunal communities include high sedimentation rates and variable freshwater inputs in estuarine systems (Puente et al. 2022) and high hydrodynamic stress in areas with dynamic tidal regimes or intense wave climates (Foulquier et al. 2020). Such dynamic environments can appear relatively unimpacted by anthropogenic influences, because contaminated water and sediments are rapidly dispersed and because communities in naturally stressed environments may be more resilient (Bolam et al. 2011; Callaway et al. 2020).

While there have been many studies on the natural and anthropogenic factors driving benthic invertebrate community patterns in coastal waters (Dutertre et al. 2013; Bae et al. 2018), there have been comparatively few focused on locations with highly energetic regimes (Foulquier et al. 2020), particularly in areas which also have long legacies of human activity (Callaway 2016; Callaway et al. 2020). Where anthropogenic influences have been considered, inferences have typically been made using proxies such as distance from potential sources of impacts (Callaway et al. 2020), rather than direct measurement of anthropogenic influence (e.g. sediment contaminant concentrations) and biological variables from the same samples.

### Rationale and Objectives of Current Study

The goal of this study was to use samples collected over a period of 10 years, from six reference sites in a strongly tidal estuary on the Canadian Atlantic coast, to gain insights into the factors driving invertebrate infaunal community characteristics in highly dynamic coastal environments. There were four principal aims: (1) to characterise spatial and temporal variation in infaunal invertebrate communities, (2) to characterise spatial and temporal variation in sediment physical properties and contaminant concentrations, (3) to apply a ‘defining normal’ approach to establish normal ranges of key variables for the sampled sites, and (4) to quantify relationships between the biological and physical/chemical data. Based on previous studies of the area, we hypothesised that contaminant levels would vary among sites but generally remain below levels of concern. Infaunal invertebrate communities were expected to vary among sites but show comparatively minor changes over the monitoring period. Biological variation was expected to be mostly related to variation in physical factors (sediment characteristics, depth).

## Methods

### Saint John Harbour

Saint John Harbour, New Brunswick, Canada, is situated at the mouth of the Saint John River, which drains 55,400 km^2^ (Marsh 2015). The harbour has a tidal range of 8 m and strong tidal currents (Delpeche 2006; Toodesh 2012), with seabed habitats ranging from muddy sediments to gravel and rock. The Port of Saint John is the largest in Atlantic Canada, with growing shipping traffic including bulk carriers, container ships, fishing vessels, LNG tankers, and cruise ships (Port Saint John 2021). Navigable channels are maintained by regular dredging, and the resulting spoil is dumped at a designated site near Black Point (Parrot et al. 2002). The metropolitan area of Saint John has a population of over 120,000; municipal wastewater is discharged into the harbour along with effluents from multiple industries including pulp and paper production, brewing, and an oil refinery. In 2010, 56% of municipal wastewater (16,000 m^3^ per day) was discharged, untreated, into rivers and creeks draining into the Harbour, but by 2014, all wastewater had been diverted to treatment facilities (City of Saint John 2014). Despite this history of intense human activity, past surveys of Saint John Harbour have noted that sediment contamination has mostly been restricted to areas relatively close to contaminant sources and that the wider harbour area is relatively unpolluted; this is usually attributed to the highly dynamic nature of the tides (Ray and Macknight 1984; Wildish and Thomas 1985).

**Fig. 1:**
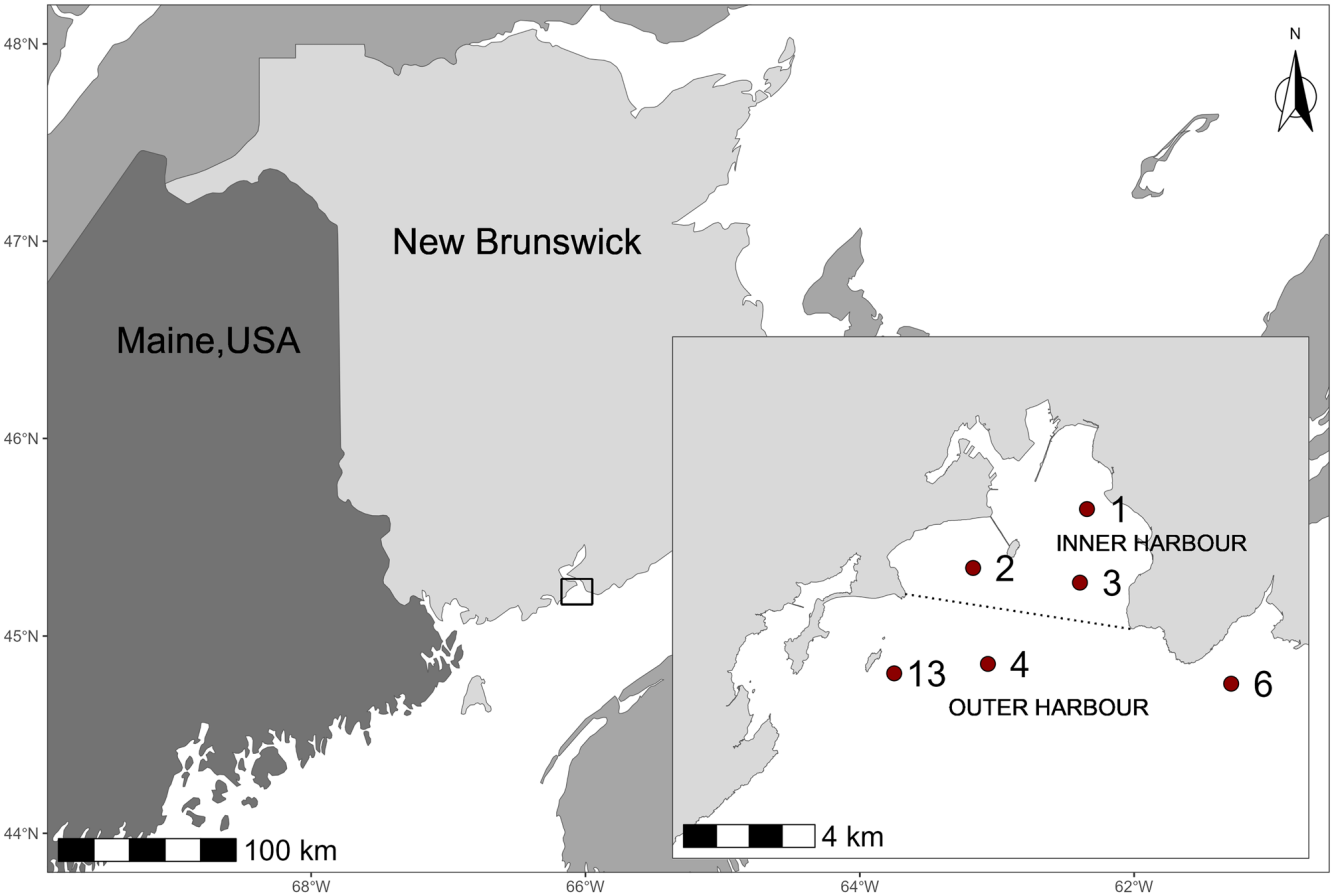
Locations of reference sites in Saint John Harbour, New Brunswick. The dotted line divides the Harbour into two regions, the Inner Harbour and the Outer Harbour. Site locations: site 1 (45.249° N, 66.025° W), site 2 (45.233° N, 66.069° W), site 3 (45.229° N, 66.028° W), site 4 (45.207° N, 66.064° W), site 6 (45.201° N, 65.969° W), site 13 (45.204° N, 66.1° W)

As part of a project to establish a long-term monitoring program, six reference sites were selected following consultations with local stakeholders and consideration of previous studies conducted in the Harbour (e.g. Envirosphere Consultants Ltd 2003; Parrot et al. 2002). Three were in the Inner Harbour, and three in the Outer Harbour (Fig. 1). Sites were chosen to be in areas of suitable soft substrate, away from known or suspected contaminant sources. These sites were also far enough out into Saint John Harbour that they were unlikely to experience reduced salinities as a result of freshwater outflow from the Saint John River; although reduced salinities have been recorded at the sea surface, the tidal salt wedge penetrates well into the Inner Harbour (Toodesh 2012) and, at locations near our sampling sites, seabed salinities appear to be fully marine at all stages of the tidal cycle (Neu 1960).

To characterise temporal patterns in the invertebrate infauna, samples were collected over 1–2 days each fall (Oct–Nov) from 2011 to 2013 and from 2017 to 2020. Additional samples were collected at other times of the year in 2011–2014 (August 2011, April and June 2012, June 2013, and June 2014). Data from these additional dates were used alongside fall data to explore correlations among metals and to determine normal ranges of these variables, as there was little evidence of strong seasonal variation (Van Geest et al. 2015), but invertebrate data from these dates were not analysed. In 2019, because of weather conditions, a slightly different location was sampled for site 6.

### Field Sampling Protocol

An ~0.1 m^2^ Smith-McIntyre grab sampler was used to collect a minimum of five replicate grab samples from each site on each sampling date. GPS position and water column depth were recorded for each sample collected. For samples where depth and exact position were not recorded, representative values for each reference site were used. Samples with a sediment penetration depth of less than 5 cm were generally rejected, although some were retained at sites where the grab had difficulty penetrating the sediment. Samples were divided into two portions, one for physical and chemical analysis and the other for infaunal analysis. The infaunal portion (surface area 0.0321 m^2^) was sieved through a 0.5 mm plastic mesh under gently flowing seawater; organisms and remaining sediments were preserved in ethanol (95% v/v purity). Pre-cleaned plastic corers were used to collect 5 cm depth, 6.4 cm diameter (~160 ml volume) subsamples from the physical/chemical portion of each grab, which were transferred to 250 ml pre-cleaned amber glass jars. For samples with a penetration depth less than 5 cm, cores were pooled to achieve an equivalent volume of sediment. Samples were kept on ice until brought ashore and frozen at –20 °C for storage.

### Processing and Analysis of Benthic Invertebrate Samples

Preserved infauna were gently sieved (0.5 mm mesh, stainless steel sieve) in freshwater to remove remaining fine sediment, and large debris was manually removed. The remaining sample portion was examined under 95% ethanol using dissecting and compound microscopes. Where individuals were fragmented during sieving (mostly polychaetes), head portions were counted for more abundant species (e.g. *Cossura longocirrata*), or head and tail portions were counted and the greater of the two numbers was recorded. Bryozoa and Foraminifera were not recorded.

Organisms were identified to species level when possible. Each new organism identified was catalogued and photographed; early in the project, this catalogue was sent to the Atlantic Reference Centre (St. Andrews, NB) to confirm species identifications. Where individuals could not be identified to species level, they were counted at higher taxonomic levels or excluded where damage made identification impossible. Where there was uncertainty regarding identification, species were merged at higher taxonomic levels (e.g. *Ampharete* spp.) and/or grouped (e.g. *Tharyx* spp. and *Chaetozone* spp.). The World Register of Marine Species (WoRMS 2022) was used to verify authoritative taxonomic names.

### Physical/Chemical Sediment Analysis

Frozen samples were thawed and homogenised, and 20 g aliquots were removed for determination of loss on ignition (LOI). The remainder of the sample was freeze-dried for at least 3 days, then homogenised using a dried, acid-washed glass mortar and pestle. Aliquots of dried sample were taken for determination of grain size, metal concentrations, total mercury, and PAHs (Supplementary Table S1).

#### Loss on Ignition, Total Organic Carbon (TOC), and Grain Size

Wet sample aliquots were weighed, dried at 105 °C for 16 h, heated at 550 °C for 3.5 h, and finally heated at 950 °C for 1.5 h, reweighing after each step. LOI_550_ and LOI_950_ were calculated using these weights. LOI_550_ was converted to TOC using an empirically determined relationship based on 20 samples analysed directly for TOC by Research & Productivity Council (RPC; Fredericton, NB) using a LECO combustion/infrared method (based on Strobel et al. 1995). Grain size determinations were based on the Folk (1980) method. Between 10 and 50 g of homogenised, dried sediment was manually shaken for 10 min (from 2011 to 2018) or on an automatic sieve shaker for 20 min (from 2019 onwards) in a sequential sieve stack (4, 1, 0.500, 0.250, 0.125, and 0.063 mm); any clumps were manually broken up. The material retained on each sieve and collected under the sieve stack was weighed, converted to percentages, and aggregated into the following categories: > 1 mm (granules and pebbles); 0.5–1 mm (coarse sand); 0.125–0.5 mm (fine and medium sand); < 0.125 mm (silt and clay).

#### Metal Concentrations

Analysis of sediments collected from 2011 to 2018 was conducted at UNB Saint John. Sample digestion and metal analysis methods were based on US Environmental Protection Agency standard testing protocols 3051A, 200.7, and 6010C. A 0.5 g aliquot of homogenised, dried sample was microwave digested in 10 ml of metal-grade nitric acid. After digestion, 40 ml of Milli-Q water and a known amount of Yttrium (Y) were added as an internal standard. Samples were filtered into polypropylene test tubes using Millex syringe filters (0.45 μm) and disposable polyethylene/polypropylene syringes. Seventeen elements (Supplementary Table S1) were quantified using an inductively coupled plasma-optical emission spectrophotometer (ICP-OES, iCAP 6500 Duo, Thermo Fisher Scientific). From 2019 to 2021, samples for metal analysis, excluding mercury, were sent to RPC (Fredericton, NB, Canada). Twenty elements (including 14 measured by UNB for 2011–2018) were measured at RPC using EPA method 3050 for sample digestion and EPA methods 200.7 and 200.8 for quantitation by ICP-ES and ICP-MS (Supplementary Table S1). Samples from 2018 were also re-analysed at RPC to allow comparison of data from the two laboratories; statistical relationships between measurements from the two laboratories were weak for most elements, making simple mathematical correction inappropriate (Supplementary Table S2). As such, metal data were analysed separately for each laboratory. Total mercury was measured separately and consistently over time, following a test method based on US EPA standard testing protocol 7473; a 0.03 g aliquot of homogenised, dried sample was run on a direct mercury analyser (Milestone DMA-80). For additional details, including QA/QC protocols, consult Van Geest et al. (2015).

#### Polycyclic Aromatic Hydrocarbons

Sample extraction and analysis of PAHs followed a test method based on US EPA standard testing protocols 3545A, 3640A, and 8270C. A minimum 10 g aliquot of homogenised, dried sample was extracted with distilled-in-glass (DIG) grade, 50:50 dichloromethane (DCM) to hexane. Extracted samples were concentrated in 50:50 DCM to hexane and run through a gel permeation column (J2 Scientific Automated Gel Permeation System) using 50:50 DCM to hexane to remove heavier contaminants and then concentrated into 1.0 ml isooctane. A standardized amount of internal standard solution (naphthalene-d8, acenaphthene-d10, phenanthrene-d10, chrysene-d12, and perylene-d12) was added to each sample prior to quantification. Concentrated extracts were run on a gas chromatograph-mass spectrometer (Agilent 6890/5975B GC–MS) and quantified using internal standard calibration and single-ion monitoring mode. Sixteen PAHs were quantified (Supplementary Table S1c), with a method detection limit (MDL) of < 0.01 mg/kg dw for individual PAHs. Data were summed for congeners above the MDL to give total PAHs (MDL < 0.04 mg/kg). Sediment PAHs were not analysed from 2018 to 2020.

### Data Analysis

R version 4.1.0 (R Development Core Team 2021) was used for all univariate analyses of biological data and all analysis of physical and chemical data. PRIMER 7.0.21 with PERMANOVA+ (Clarke and Gorley 2015) was used for multivariate biological analyses.

#### Infauna

Only fall (October/November) invertebrate data were analysed (219 samples), as previous analyses identified strong seasonal variation in biological communities (Van Geest et al. 2015). Four univariate indices were calculated: total abundance, species richness, Shannon diversity (*H*), and AZTI Marine Biotic Index (AMBI: Borja et al. 2000), the latter calculated using AMBI v6.0 (species list updated December 2020). Where a species was not present in the AMBI database, the score for a suitable congener was used (4.3% of species) or the species was assigned the score for a higher taxonomic level (12.8% of species); if this was not possible, or if congeners had a range of different ecological group (EG) scores, the species was retained but marked as ‘not assigned’ (5.5% of taxa). As AMBI was originally conceived and formulated for a European context, modified EG scores for North American marine environments provided in Gillett et al. (2015) were used where appropriate (6.7% of species). Overall abundance, species richness, Shannon diversity, and AMBI were compared among sites and years using two-way crossed ANOVA with site and year as fixed factors. To assess potential changes over time at each site, samples from early years (2011–2013) and later years (2017–2020) were compared using ANOVA with planned contrasts; similarly, Inner and Outer Harbour sites were compared for each year using planned contrasts. AMBI scores were not calculated for some samples from site 1 (most samples from 2019, some from 2018 and 2017), as AMBI can suffer from reduced robustness for samples with low numbers of taxa or individuals (Borja and Muxika 2005). Site 1 was therefore not included in the ANOVA for AMBI. Abundance data departed substantially from ANOVA assumptions due to extremely high abundances in samples from sites 2 and 3 in 2011. 2011 data were therefore excluded from this analysis, and the remaining data were square-root transformed. Within each ANOVA, *p* values for planned contrasts were adjusted to account for multiple comparisons, using the Benjamini and Hochberg method (Benjamini and Hochberg 1995).

Multivariate analysis used the Bray-Curtis similarity measure, calculated on fourth-root transformed abundance data. Centroids for each site in each year (site–year centroids) were also calculated and used to compute a second resemblance matrix. Relationships among samples and among site–year centroids were visualised using non-metric multi-dimensional scaling (nMDS). Assemblage data were analysed using two-way PERMANOVA with site and year as fixed factors, followed by pairwise PERMANOVA and PERMDISP to examine patterns among factor levels, after adjustment of *p* values as described above. BVSTEP was used to identify the minimal subset of species capable of reproducing the overall multivariate pattern in the full dataset, alongside SIMPER analysis to identify additional species differentiating site/year combinations.

#### Physical/Chemical Variables

For characterisation of the physical and chemical environment and to examine relationships between different physical/chemical variables, data from all sampling dates were used (423 samples). Measurements below relevant detection limits were replaced with randomly generated values between 0.005 and the detection limit. Metal data were explored using principal component analysis (PCA) on data from all sampling dates, separately for UNB and RPC data. Since samples were analysed consistently over time for mercury, analysis of mercury data used all dates except for 2017, when mercury was not measured.

#### Relationships Between Physical/Chemical Variables and Infauna

Relationships between univariate invertebrate community metrics and physical/chemical variables were modelled by partial least squares regression (PLSR) using the *pls* package (Mevik and Wehrens 2007), including only data from fall samples with corresponding physical/chemical and biological data (211 samples). Since UNB and RPC metal data could not be combined, UNB metal data for 2011–2013 and 2018 were used; 2017 was excluded as mercury and grain size data were unavailable. PAHs were not included as they were not measured in 2018. Potential explanatory variables were therefore metal concentrations (including mercury), grain size characteristics, TOC, LOI950, and water column depth. For the abundance model, 2011 data from sites 2 and 3 were excluded as these had atypically high values. Two samples from site 1 in 2018 were excluded from the AMBI model because of very low abundances. Although PLSR is intended to account for autocorrelation, where physical or chemical variables were highly correlated (Pearson’s *ρ* ≥ 0.95), one was selected and others were excluded. Percent fine/medium sand was excluded as it was negatively correlated with percent silt/clay (Pearson’s *ρ* = −0.97, *p* < 0001); vanadium was excluded as it was correlated with chromium (*ρ* = 0.95, *p* < 0.001), and cobalt and nickel were excluded because they were correlated with zinc (*ρ* = 0.95/0.96 respectively, *p* < 0.001). Most variables were log-transformed to reduce right skew, excluding Cu, P, and Rb, while percent silt/clay data were inverse log-transformed: SiltClay_transformed_ = 5 − log(101 − % SiltClay). Abundance data were log-transformed prior to analysis to reduce heteroscedasticity in model residuals. The number of PLSR components used in the final models was selected based on minimisation of root mean square prediction error in leave-one-out cross-validation. Relative importance of each predictor variable in each of the PLSR models was assessed using variable influence in projection (VIP) scores (Wold et al. 2001; Chong and Jun 2005) obtained using the *mixOmics* package (Rohart et al. 2017).

DistLM was used to explore relationships between physical/chemical variables and the multivariate invertebrate data, identifying the best models using stepwise selection with AICc as the optimisation criterion. Contaminant metals were grouped such that they were selected (or not) together, excluding La, P, and Rb, which did not have similar component loadings in exploratory PCA.

#### Determining ‘Normal’ Ranges for Saint John Harbour Baseline

For key physical and chemical variables and univariate biological indices, ‘normal ranges’ of variation were calculated (Arciszewski and Munkittrick 2015). Ranges were calculated at three levels: Site, Inner or Outer Harbour, and Saint John Harbour as a whole. Individual site normal ranges were defined as being within ± 2 standard deviations of the grand mean for that site, calculated using the mean values for each sampling date. Inner Harbour (sites 1–3) and Outer Harbour (sites 4, 13, and 6) ranges and overall Saint John Harbour (all sites) ranges were also calculated (Supplementary Table S3).

## Results

**Fig. 2:**
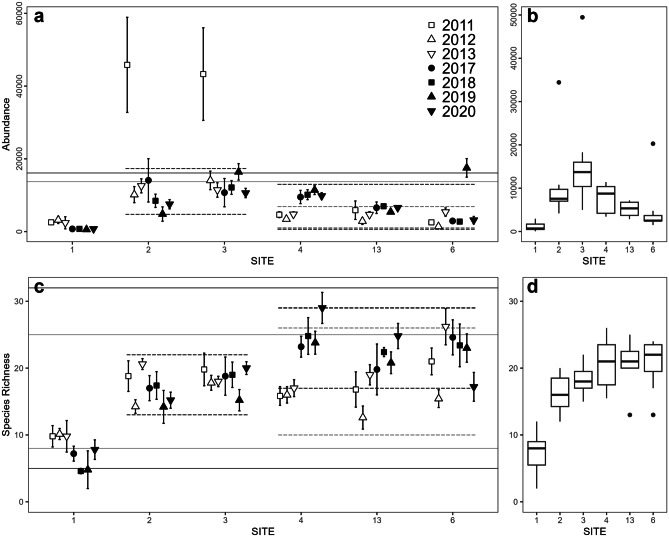
**a** Mean abundance (individuals per square meter) ± SE for each site on fall sampling dates. **b** Median abundance for each site over all years (*n* = 7). **c** Mean species richness ± SE for each site on fall sampling dates. **d** Median species richness over all years (*n* = 7). Reference lines represent normal ranges; dashed lines: Inner/Outer Harbour; solid lines: Saint John Harbour overall. For Outer Harbour and overall ranges, grey lines indicate normal ranges for 2011–2013, and black lines for 2017–2020. For further details, see Supplementary Table S3a

One hundred eighty-eight taxa were included in the infaunal data analyses. ANOVAs for abundance, species richness, Shannon diversity, and AMBI all showed significant site–year interactions (Supplementary Table S4). Total abundance of infaunal invertebrates changed at some sites (Fig. 2a). In the Outer Harbour, there was a clear increase at site 4 and a smaller increase at site 13 (Table 1). In the Inner Harbour, there was a significant but comparatively small decline at site 1 and a smaller but significant decline at site 2 against a background pattern of very high interannual variability, including abnormally high abundance in 2011. Within years, abundance also varied among sites, but did not consistently differ significantly between the Inner and Outer Harbour, since abundance tended to be both lowest (at site 1) and highest (at sites 2 and 3) within the Inner Harbour (Fig. 2a, b; Table 2).

**Table 1:** Temporal trends in abundance, species richness, Shannon diversity, and AMBI: ANOVA planned contrasts comparing early (2011–2013) and late (2017–2020) sampling years

Site	Metric	Difference (early vs. late)	df	*t* ratio	*p* (adjusted)
1	**Abundance**	**−24.34**^a^	**151**	**−3.010**	** < 0.01**
	**Species richness**	**−3.86**	**177**	**−2.675**	**0.013**
	Shannon diversity	**−**0.05	177	**−**0.470	0.693
2	**Abundance**	**−17.91**^a^	**151**	**−2.252**	**0.044**
	Species richness	**−**1.93	177	**−**1.350	0.258
	Shannon diversity	**0.51**	**177**	**4.803**	** < 0.001**
	No 2011^b^	0.17^b^	177^b^	1.449^b^	0.194^b^
	AMBI	**−**0.24	147	**−**1.565	0.144
3	Abundance	**−**2.48^a^	151	**−**0.291	0.841
	Species richness	**−**0.28	177	**−**0.191	0.849
	Shannon diversity	**0.36**	**177**	**3.303**	**0.003**
	No 2011^b^	0.24^b^	177^b^	1.907^b^	0.097^b^
	**AMBI**	**−0.52**	**147**	**−3.278**	**0.0019**
4	**Abundance**	**37.24**^a^	**151**	**4.381**	** < 0.001**
	**Species richness**	**8.92**	**177**	**6.103**	** < 0.001**
	Shannon diversity	0.21	177	1.897	0.086
	AMBI	0.22	147	1.407	0.176
13	**Abundance**	**18.42**^**a**^	**151**	**2.360**	**0.044**
	**Species richness**	**5.82**	**177**	**3.915**	** < 0.001**
	Shannon diversity	**−**0.02	177	**−**0.197	0.844
	AMBI	**−**0.02	147	**−**0.144	0.886
6	**Abundance**	**20.07**^**a**^	**151**	**2.277**	**0.044**
	No 2019^c^	**−**1.26^c^	151^c^	**−**0.148^c^	0.8829^c^
	Species richness	1.23	177	0.866	0.458
	Shannon diversity	**−**0.14	177	**−**1.301	0.230
	AMBI	**−**0.31	147	**−**1.979	0.066

Site 1 not included in the AMBI model; 2011 data not included in the abundance model. Bold format indicates significant difference (*p* < 0.05). *p* values have been adjusted (within each variable) to account for multiple comparisons using the Benjamini and Hochberg (1995) method

^a^Estimates for abundance are in the model scale (square-root transformed data)

^b^Alternative estimates for sites 2 and 3 if 2011 data are excluded

^c^Alternative estimates for site 6 if 2019 data are excluded

**Table 2:** Spatial differences in abundance, species richness, Shannon diversity, and AMBI: ANOVA planned contrasts comparing Inner (1–3) and outer (4, 13, 6) Harbour sites

Year	Metric	Difference (Inner vs. Outer)	df	*t* ratio	*p* (adjusted)
2011	Species richness	1.69	177	1.094	0.358
	**Diversity**	**1.12**	**177**	**9.774**	**< 0.001**
	**AMBI**	**−2.07**	**147**	**−11.23**	**< 0.001**
2012	**Abundance**	**−7.23** ^a^	**151**	**−5.565**	**< 0.001**
	Species richness	0.61	177	0.420	0.731
	**Diversity**	**0.63**	**177**	**5.853**	**< 0.001**
	**AMBI**	**−2.03**	**147**	**−11.69**	**< 0.001**
2013	Abundance	**−**2.98 ^a^	151	**−**2.106	0.055
	**Species richness**	**4.60**	**177**	**2.896**	**0.008**
	**Diversity**	**0.29**	**177**	**2.473**	**0.031**
	**AMBI**	**−1.65**	**147**	**−8.711**	**< 0.001**
2017	Abundance	**−**0.272 ^a^	151	**−**0.192	0.848
	**Species richness**	**8.20**	**177**	**5.162**	**< 0.001**
	**Diversity**	**0.38**	**177**	**3.246**	**0.005**
	**AMBI**	**−1.44**	**147**	**−7.619**	**< 0.001**
2018	Abundance	0.820 ^a^	151	0.579	0.676
	**Species richness**	**9.87**	**177**	**6.212**	**< 0.001**
	**Diversity**	**0.76**	**177**	**6.447**	**< 0.001**
	**AMBI**	**−1.72**	**147**	**−9.108**	**< 0.001**
2019	**Abundance**	**6.24 **^**a**^	**151**	**4.407**	**< 0.001**
	No Site 6	**−**0.223 ^ab^	151	**−**0.148	0.883
	**Species richness**	**11.20**	**177**	**7.051**	**< 0.001**
	**Diversity**	**0.34**	**177**	**2.838**	**0.013**
	**AMBI**	**−1.74**	**147**	**−9.214**	**< 0.001**
2020	Abundance	1.159	151	0.819	0.414
	**Species richness**	**9.33**	**177**	**5.876**	**< 0.001**
	Diversity	0.21	177	1.803	0.106
	**AMBI**	**−1.41**	**147**	**−7.480**	**< 0.001**

Bold format indicates significant difference (*p* < 0.05). *p* values have been adjusted (within each variable) to account for multiple comparisons using the Benjamini and Hochberg (1995) method. Site 1 not included in AMBI model; 2011 data not included in Abundance model

^a^Estimates for abundance are in the model scale (square-root transformed data)

^b^Alternative estimates for 2019 if site 6 data are excluded

Species richness changed over time at some sites (Fig. 2c), particularly in the Outer Harbour, where it increased at two of three sites (4 and 13); but there was little evidence of directional change in the Inner Harbour aside from a small decline at site 1 (Table 1). Richness appeared to be lowest at site 1 on all sampling dates, although in 2011–2012, there were no significant differences between Inner and Outer Harbour sites (Table 2; Fig. 2c, d). From 2013 onwards, richness was significantly higher in the Outer Harbour, most likely because of the above-mentioned increases at two of these sites (sites 4 and 13).

**Fig. 3:**
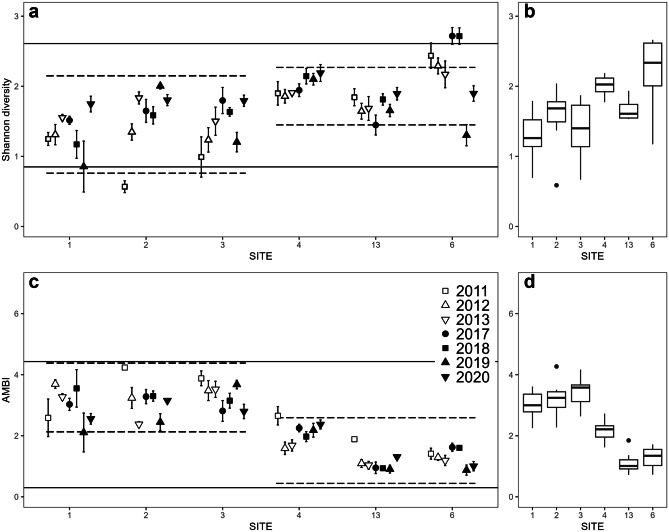
**a** Mean Shannon diversity ± SE for each site on fall sampling dates. **b** Median diversity for each site over all years (*n* = 7). **c** Mean AMBI ± SE for each site on fall sampling dates. **d** Median AMBI for each site over all years (*n* = 7). 2019 data from site 1 and some individual samples from other years were excluded from AMBI plots (see ‘Methods’). Reference lines represent normal ranges; dashed lines: Inner/Outer Harbour; solid lines: Saint John Harbour overall. For further details, see Supplementary Table S3a

There was little evidence that Shannon diversity changed significantly over time (Table 1), despite clear interannual variability at most sites (Fig. 3a). Diversity was significantly higher in the Outer Harbour in all years apart from 2020 (Table 2, Fig. 3b), particularly at site 6. While there was interannual variation in AMBI at all sites, there was little evidence of directional trends (Fig. 3c, Table 1). Mean AMBI was significantly higher in the Inner Harbour in all years (Table 2; Fig. 3c, d); normal ranges of Inner Harbour AMBI values correspond to slightly disturbed/disturbed condition categories while Outer Harbour ranges correspond to undisturbed/slightly disturbed conditions (Supplementary Table S3).

### Multivariate Patterns in Infaunal Invertebrate Data

**Fig. 4:**
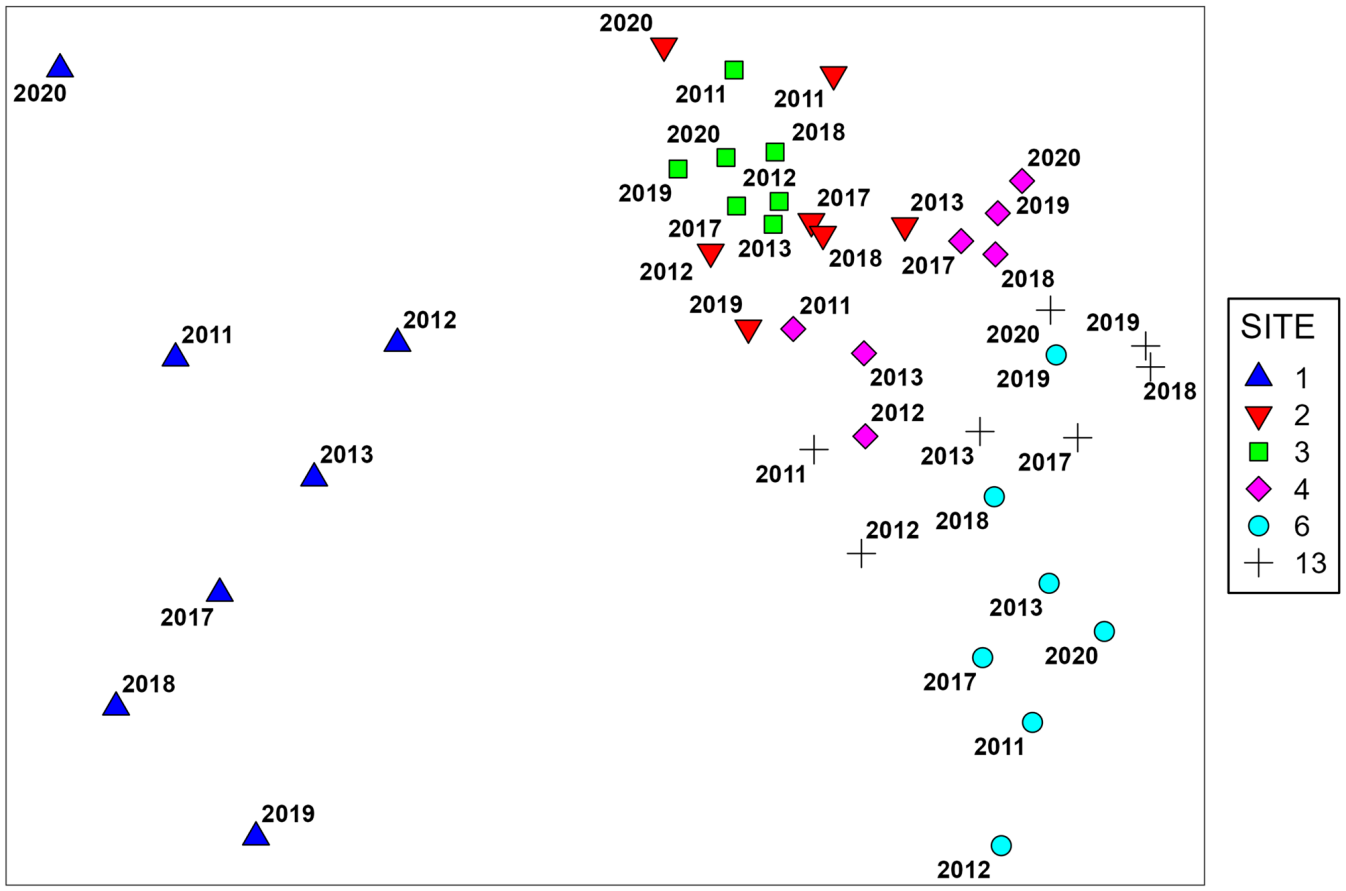
nMDS ordination of centroids calculated for each site in each year (Bray-Curtis similarity, fourth-root transformed abundance data). Stress = 0.1 For ordination of individual sample data, see Supplementary Fig. S1

There was obvious variation in invertebrate community composition among sites (nMDS: Fig. 4, PERMANOVA: Table 3); differences among sites varied over time, and temporal trends were not consistent across all sites (significant site–year interaction). Pairwise PERMANOVA comparisons within years showed that each site was distinct from all other sites in all years, except for Inner Harbour sites 2 and 3, which only differed significantly from each other in 2011 and 2013 (Supplementary Table S5). Inner Harbour site 1 was very distinct in composition, with centroids and samples well separated from all other sites (Fig. 4, Supplementary Fig. S1), while sites 2 and 3 were similar, with overlapping samples and centroids. In the Outer Harbour, there was little or no overlap between sites 4 and 6, while site 13 appeared intermediate, overlapping with both. Multivariate dispersion also varied significantly among sites in all years except 2013 (PERMDISP; Table 3). While PERMANOVA can be sensitive to significant variation in dispersion (Anderson and Walsh 2013), the sites which tended to have significantly greater dispersion (sites 1 and 6) were also the most distinct (Fig. 4, Supplementary Fig. S1), so it is likely that the significant differences in PERMANOVA represent genuine differences in composition.

**Table 3:** Summary of PERMANOVA and PERMDISP analyses

	PERMANOVA global test				
Source	df	SS	MS	Pseudo-*F*	*p* (perm)
Site	5	126,750	25,349	26.24	**< 0.001**
Year	6	30,403	5068	5.25	**< 0.001**
Site*year	30	63,898	2130	2.21	**< 0.001**
Residual	177	170,960	966		
Total	177	393,190			

PERMDISP – sites (within years)	*F*	df	*p* (perm)		PERMDISP – years (within sites)	*F*	df	*p* (perm)
2011	4.77	5.26	**0.026**		Site 1	5.16	6.30	**0.015**
2012	6.65	5.31	**0.003**		Site 2	4.13	6.31	**0.043**
2013	3.28	5.24	0.098		Site 3	0.95	6.28	0.706
2017	4.80	5.24	**0.047**		Site 4	9.68	6.29	**< 0.001**
2018	9.11	5.24	**< 0.001**		Site 13	2.78	6.28	0.189
2019	23.1	5.24	**< 0.001**		Site 6	5.00	6.31	**< 0.001**
2020	5.05	5.24	**0.018**					

Full PERMANOVA results, including pairwise comparisons, can be found in Supplementary Table S5

**Fig. 5:**
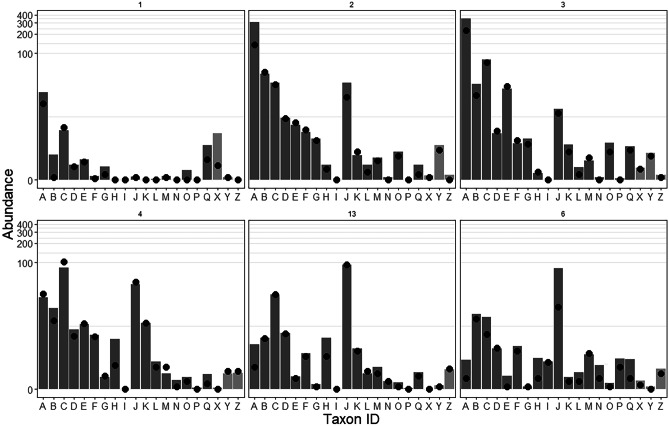
Mean abundance (bars) and median abundance (points) of 17 taxa identified by BVSTEP (A–Q) and three additional taxa identified from SIMPER analyses (X–Z). Replicates are means on each date (*n* = 7). Taxa A–I: Polychaeta; taxa J–M: Mollusca; taxa N–P: Crustacea; taxon Q: Nematoda. Additional taxa; X: *Clymenella torquata* and other Maldanidae (excluding *Rhodine* spp.), Polychaeta; Y: *Eteone longa*, Polychaeta; Z = *Thyasira flexuosa*, Mollusca, Bivalvia. Note non-linear (‘pseudo-logarithmic’) *y*-axis scale. For full key to taxon ID, means, and variability, see Supplementary Table S6; for temporal trends, see Supplementary Figs. S2–S7

BVSTEP identified 17 taxa sufficient to replicate the full multivariate data pattern, and several additional taxa were identified using SIMPER as relevant for specific comparisons among years and sites (Fig. 5, Supplementary Table S6). Abundances of most of these taxa were low at Inner Harbour site 1, and molluscs in particular were almost absent. Some trends in polychaete species were notable at site 1: *Clymenella torquata* was more abundant than at the other sites; *Capitella capitata* (an opportunist; Grassle and Grassle 1974) was present at low abundances in all years except 2012 (it was only occasionally recorded at the other sites); and *Micronephthys neotena* was recorded, but only in 2020. Inner Harbour sites 2 and 3 were distinguished from the Outer Harbour sites by generally higher abundances, particularly of key polychaete species *Cossura longocirrata*, *Levinsenia gracilis*, *Tharyx/Chaetozone*, *Sternaspis scutata*, and *Eteone longa.* However, abundances of other taxa were lower at these sites compared to the Outer Harbour, including the bivalve *Nucula proxima* and the polychaete *Terebellides stroemii*. The polychaete *Micronephthys neotena* was recorded at both Inner Harbour sites from 2017 onwards in increasing numbers. In the Outer Harbour, site 6 had a distinct species composition; the gastropod *Ilyanassa trivittata*, the cumacean *Eudorella truncatula*, and the amphipod *Unciola irrorata* were more abundant here than at other sites, and the polychaete *Sabellaria vulgaris*, almost completely absent from all other sites, was present. Several polychaete species, particularly *Cossura longocirrata* and *Tharyx/Chaetozone* (both opportunists; Olsgard and Hasle 1993), had lower abundances at site 6 than at all other sites except site 1.

Communities in the early years (2011–2013) tended to be distinct from those in the later years (2017–2020), but this was not the case at all sites (PERMANOVA; Table 3, Supplementary Table S5). The pattern was clearest in the Outer Harbour, with centroids for early and late years forming separate clusters at sites 4 and 13 (Fig. 4). Changes at these sites were related to increases in overall abundance and richness (Fig. 2) and abundances of several individual taxa (Supplementary Figs. S5 and S6) in 2017–2020. In contrast, at site 6, there was no clear separation into early vs. late years and little evidence of consistent change in any species (Supplementary Fig. S7). In the Inner Harbour, site 1 changed between 2011–2013 and 2017–2019 (Fig. 4), largely reflecting declines in abundance of several species (Supplementary Fig. S2), particularly the polychaetes *Cossura longocirrata*, *Tharyx/Chaetozone*, and *Nephtys incisa*. Patterns were less clear for Inner Harbour sites 2 and 3; early years were significantly different from later years, but there were also significant differences among the early years. In particular, 2011 differed from all other years at both sites, driven mainly by higher abundances of the polychaetes *Cossura longocirrata* and *Rhodine loveni*.

### Sediment Physical and Chemical Characteristics

**Fig. 6:**
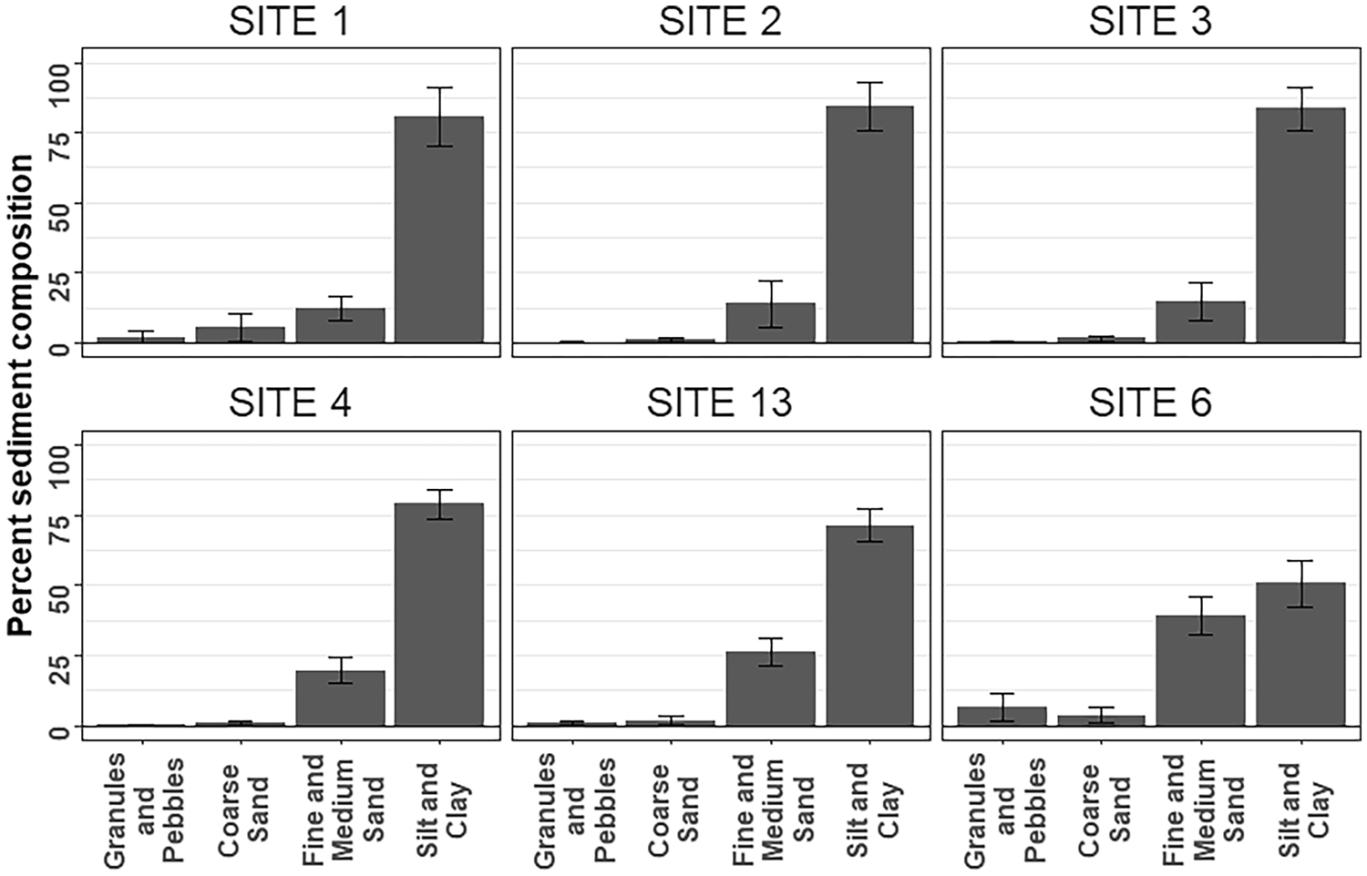
Mean sediment grain size composition across all sampling dates (*n* = 11, except site 13, *n* = 10), ± 95% confidence intervals. No grain size data were available for 2017

**Fig. 7:**
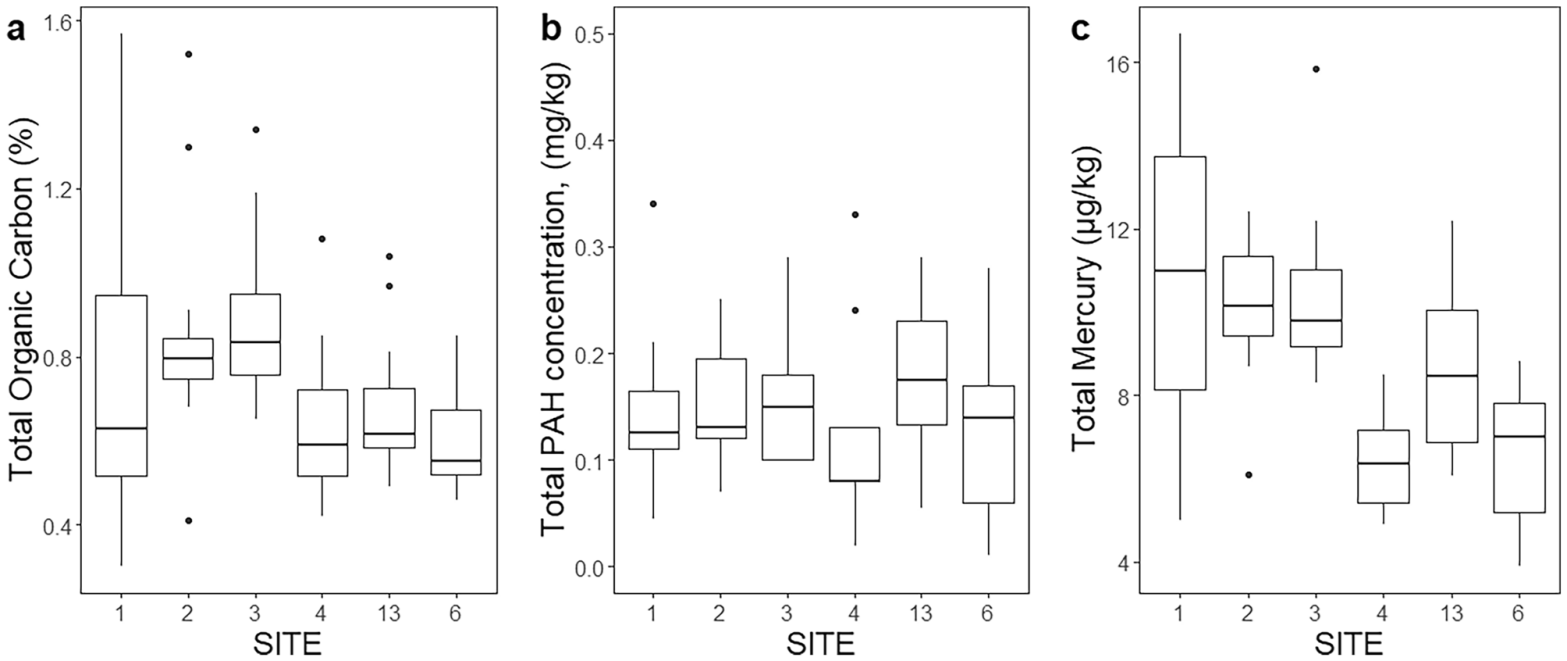
Selected chemical properties of sediment samples from reference sites, across all sampling dates. Replicates are mean values for each date. **a** Total organic carbon: *n* = 12 (11 for site 13). **b** Total PAH: *n* = 9 (8 for site 13); one outlier has been excluded for site 1 (November 2013, mean TPAH = 2.28 mg/kg). **c** Total mercury: *n* = 11 (10 for site 13). For data from individual sampling dates, see Supplementary Figs. S8–S10

Sediments were predominantly silt and clay (grain size < 0.125 mm), particularly in the Inner Harbour (Fig. 6). Inner Harbour sites (1–3) had similar grain size distributions, although site 1 tended to have slightly more of the coarser fractions. In comparison, in the Outer Harbour, sites 4 and 13 had more fine and medium sand (0.125–0.5 mm) and less silt and clay, while site 6 was distinct, with a considerably lower proportion of silt and clay (approximately 50%) and > 35% fine and medium sand. Total organic carbon was generally low, ranging from 0.18 to 1.74%, with little or no indication of temporal trends at any of the sites (Supplementary Fig. S8). TOC tended to be higher for the Inner Harbour sites (Fig. 7a), but differences were small, and variability among sampling dates was relatively high, particularly at site 1.

**Fig. 8:**
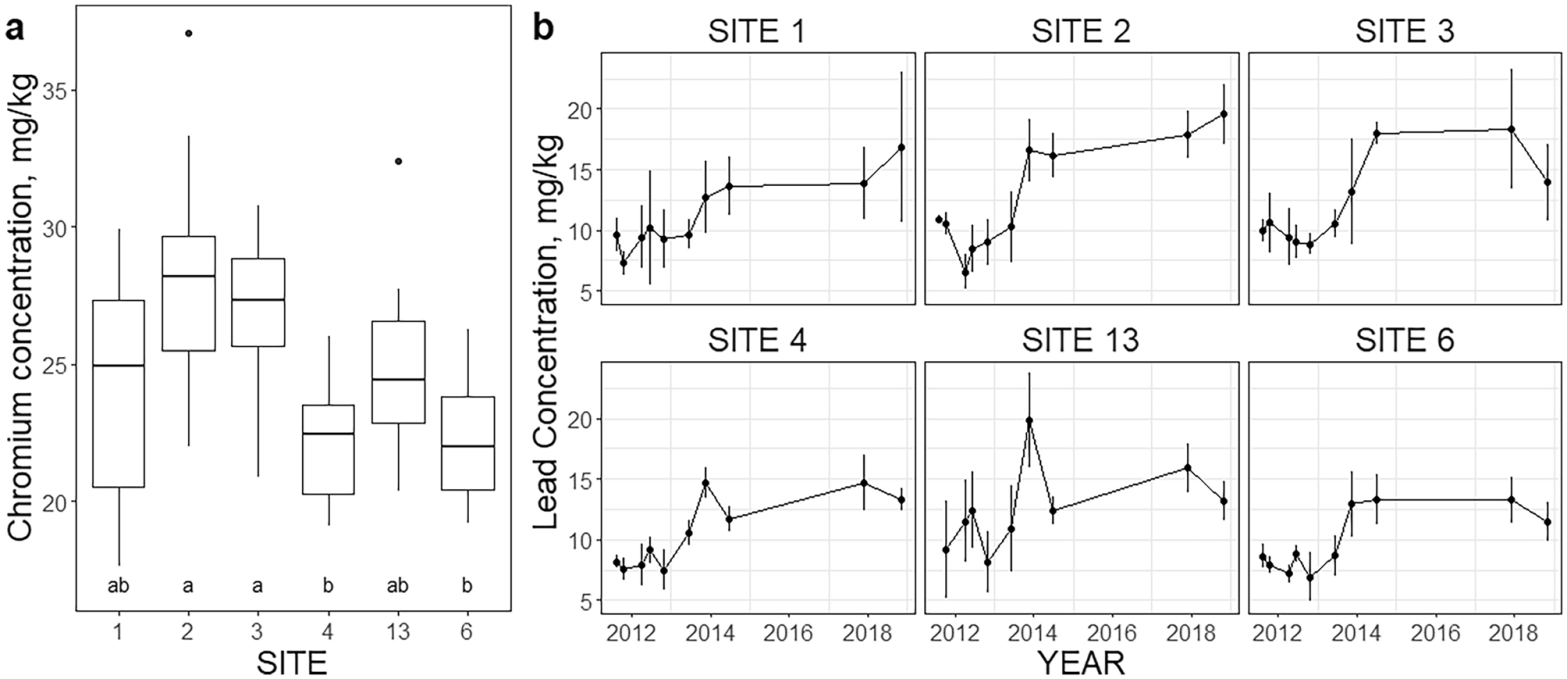
**a** Chromium concentration, representative of principal component 1 from PCA on UNB metal data (2011–2018), *n* = 10 (9 for site 13). Bars marked with different letters are significantly different (*p* < 0.05) in Tukey HSD pairwise tests following significant (*p* < 0.05) ANOVA. **b** Mean lead concentrations at the six reference sites from 2011 to 2018 (all sample dates). Error bars are ± 95% confidence intervals

PAH concentrations were low and not particularly variable among sites (Fig. 7b) or over time (Supplementary Fig. S9). Only 4 samples exceeded the interim sediment quality guideline (ISQG) level of 1.684 mg/kg for total PAH (CCME 1999), all from Inner Harbour site 1 in November 2013. Sediment quality guidelines were also available for nine metals (CCME 1999; Buchman 2008). Of these, only arsenic and nickel had recorded concentrations exceeding the ISQG/threshold effect level (TEL), and none exceeded the higher probable effect level (PEL) threshold (Table 4; Supplementary Table S4d). Mercury concentrations appeared slightly higher in the Inner compared to the Outer Harbour (Fig. 7c), but there was little indication of increases or decreases over time (Supplementary Fig. S10). The first three principal components from the PCA for all UNB trace metal data (2011–2018) explained 77.5% of the total variance (Supplementary Table S7). PC1 corresponded to general metal contamination, with most metals having similar loadings apart from Pb, La, and P. Metal contamination (exemplified by chromium, Fig. 8a) was higher for Inner Harbour sites 2 and 3 compared with Outer Harbour sites 4 and 6; sites 1 and 13 were intermediate. There were no clear temporal trends in PC1 at any site (Supplementary Fig. S11). PC2 had high negative loadings of La and P, while PC3 was dominated by high positive loading of Pb; there was little variation among sites in either component, although samples from site 6 had significantly lower La concentrations (Supplementary Fig. S12). PC2 and PC3 both increased over time at all sites (Supplementary Fig. S11), reflecting apparently higher lead concentrations from 2014 onwards (Fig. 8b). PCA of RPC metal data (2018–2021) gave similar results (Supplementary Table S8). PC1 alone explained 84.6% of the variation in the RPC metal data, with similar loadings of all metals except for Ca and Ba. There were no clear temporal trends and no indication of continued increases in Pb (Supplementary Fig. S13).

**Table 4:** Normal ranges (± 2SD from mean for Saint John Harbour) for metals with available threshold effect levels (TEL)/interim sediment quality guidelines (ISQG) and probable effect levels (PEL)

				UNB data (2011–2018)		RPC data (2018–2021)	
Metal	Units	TEL (ISQG)	PEL	Normal range	Max	Normal range	Max
Ag^a^	mg/kg	0.73	1.77		0.61		
**As**	**mg/kg**	**7.24**	**41.6**	**4.45–9.01**	**14.9**	**3.09–7.24**	**11**
Cd^a^	mg/kg	0.7	4.2		0.21		
Cr	mg/kg	52.3	160	16.7**–**32.8	42.9	11.5**–**24.8	31
Cu	mg/kg	18.7	108	2.3**–**11.9	17.1	5.09**–**12.5	18
**Ni**	**mg/kg**	**15.9**	**42.8**	**11.9–22.7**	**31.3**	**11.2–23**	**31**
Pb	mg/kg	30.24	112	4.69**–**18.3	24.1	6.74**–**14.2	19.4
Zn	mg/kg	124	271	29.6**–**59.1	89.4	30.2**–**57.2	73
Hg^b^	μg/kg	130	700	2.98**–**15.6	41		

Ranges are calculated separately for UNB and RPC metal data. Metals which exceeded the TEL/ISQG at reference sites are highlighted in bold. For site-specific ranges for all metals, see Supplementary Table S4d

^a^Normal ranges for silver and cadmium not calculated: high proportions of measurements were below detection limits

^b^Mercury concentrations were measured separately from other metals; only one normal range is presented, including all data from 2011 to 2020

### Linkage of Physical/Chemical and Invertebrate Data

**Fig. 9:**
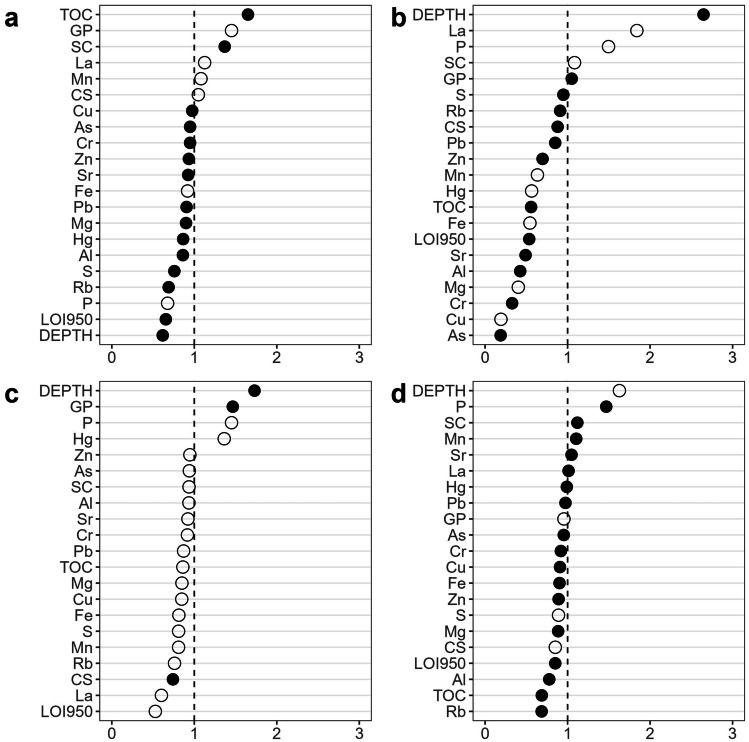
Variable influence in projection (VIP) scores for physical and chemical variables included in univariate PLSR models for **a** total abundance, **b** species richness, **c** Shannon diversity, and **d** AMBI. Generally, variables with VIP < 1 (dashed line) are not considered influential predictors. Symbol fill indicates direction of predictor-response variable relationship: filled symbols = positive relationship; open symbols = negative relationship. GP = percent granules and pebbles; SC = percent silt and clay, and CS = percent coarse sand

For the univariate community indices, the best models used the first two to five PLSR components (Supplementary Table S9), explaining 44.95% of the variance in species richness, 48.76% of the variance in total abundance, 57.09% of the variance in Shannon diversity, and 65.38% of the variance in AMBI. Depth was the most important variable influencing richness, diversity, and AMBI, but was not an important predictor of abundance (Fig. 9). Total abundance of invertebrates was most strongly positively linked with organic carbon and fine sediment content, while higher species richness and diversity (and lower AMBI) were associated with lower proportions of finer grained sediments (lower percent silt and clay, higher percent granules and pebbles). Metal concentrations were generally not among the more important predictors (Fig. 9), although higher mercury concentrations were associated with lower Shannon diversity and (more weakly) with higher AMBI. AMBI and total abundance appeared to be more sensitive to metal concentrations; a few metals had VIPs > 1, and the median VIP for all metals was 0.919 in both the abundance and AMBI models, compared to 0.870 for Shannon diversity and 0.564 for species richness.

**Fig. 10:**
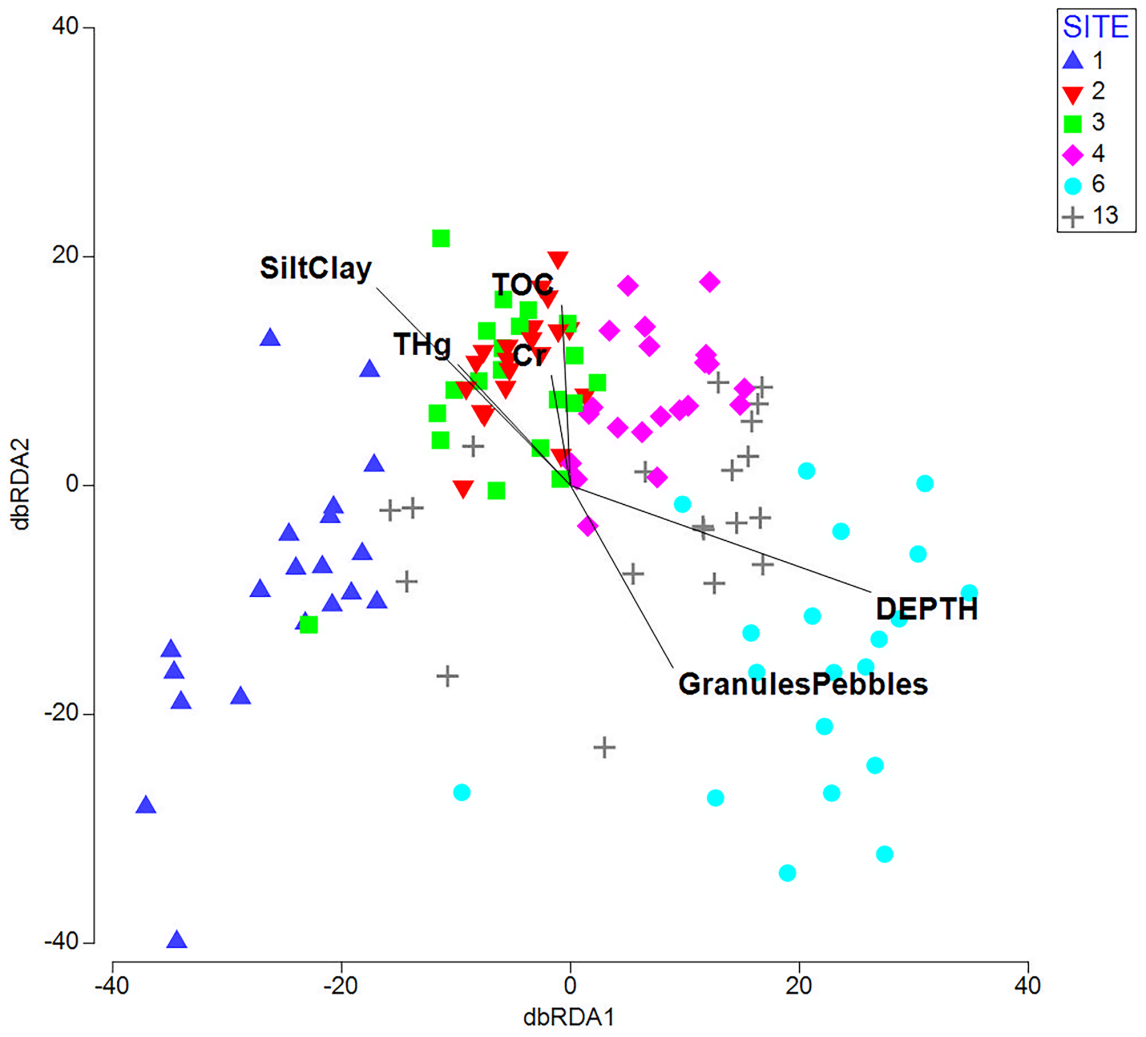
Distance-based redundancy analysis ordination based on the best DistLM model for the relationship between physical/chemical variables and infaunal community composition data. Vector overlay indicates strength of correlations with key variables of interest: Depth, total organic carbon (TOC), percent silt and clay (SiltClay), percent granules and pebbles (GranulesPebbles), chromium concentration (Cr), and mercury concentration (THg). dbRDA axes 1 and 2 explain 37.03 and 23.56% of fitted variation (15.5 and 9.86% of total variation)

The best DistLM model explained 41.86% of the variance in the multivariate community data and included depth, metals (excluding phosphorus and rubidium), lanthanum, percent silt/clay, and percent granules/pebbles (Table 5). All ten of the overall best solutions included depth, percent silt/clay, and percent granules/pebbles. Mostly importantly, these latter variables were those most strongly correlated with the dbRDA axes; correlations with concentrations of contaminants were much weaker, indicating that these were not important predictors (Fig. 10).

**Table 5:** Best 10 models selected by stepwise DISTLM

AICc	*r*^2^	Variables included
872.42	0.4186	Depth, metals*, La, %silt/clay, %granules/pebbles
872.92	0.4162	Depth, metals*, TOC, %silt/clay, %granules/pebbles
872.97	0.4291	Depth, metals*, La, TOC, %silt/clay, %granules/pebbles
873.15	0.4018	Depth, metals*, %silt/clay, %granules/pebbles
873.27	0.2939	Depth, La, P, TOC, LOI950, %silt/clay, %granules/pebbles
873.32	0.4275	Depth, metals*, La, LOI950, %silt/clay, %granules/pebbles
873.43	0.4137	Depth, metals*, LOI950, %silt/clay, %granules/pebbles
873.45	0.2793	Depth, P, TOC, LOI950, %silt/clay, %granules/pebbles
873.47	0.2792	Depth, La, P, LOI950, %silt/clay, %granules/pebbles
873.66	0.2916	Depth, La, P, Rb, LOI950, %silt/clay, %granules/pebbles

^*^Metals include Al, As, Cr, Cu, Fe, Mg, Mn, Pb, S, Sr, Zn, and Hg, grouped such that they were selected/deselected together. La, P, and Rb were included separately, as they did not have similar loadings in exploratory PCA. All of these models have AIC values within 2 AIC of the ‘best’ model and may be considered to have equivalent predictive power

## Discussion

### Drivers of Invertebrate Community Characteristics

The most important predictors for the univariate biological indices and the multivariate community data from the Saint John Harbour reference sites tended to be physical factors: water depth and sediment grain size measures. While these factors have strong relationships with infaunal invertebrate community properties (Glockzin and Zettler 2008), both are thought to be proxies for unmeasured factors, rather than necessarily having a direct influence (Snelgrove and Butman 1994; McArthur et al. 2010; Jordà Molina et al. 2019). Other factors, some of which are linked to depth and sediment properties, are also important. Sediment deposition and erosion influence infaunal communities through effects on food supply, stability of seabed habitats, and sediment grain size distribution. Deposition and erosion are in turn strongly influenced by hydrodynamic conditions, which can also exert a direct influence on biological communities (Warwick and Uncles 1980; Foulquier et al. 2020), for example, via effects on food supply and feeding behaviours (Miller et al. 1992; Roegner 1998). A previous study of the invertebrate fauna of Saint John Harbour identified an area of comparatively impoverished fauna, which was attributed to a strong tidally driven erosion–deposition cycle (Wildish and Thomas 1985). Site 1 lies within this area, so the comparatively low richness and abundance of infauna at this site could be explained by this natural stressor (Kröncke et al. 2018), rather than by sediment contamination, which did not differ substantially from sites 2 and 3. The near absence of the suspension-feeding bivalve molluscs *Nucula proxima*, *Ennucula delphinodonta*, and *Kurtiella planulata* from site 1 is also consistent with this explanation. Salinity might explain some of the remaining variation among sites, given the high outflow of freshwater from the Saint John River. However, while opportunistic measurements taken during sampling detected reduced surface salinity at some sites on some dates (data not shown), previous studies suggest that a salinity gradient is unlikely at the seabed at sampled sites, given the strong tidal mixing in the harbour and the penetration of the salt wedge well into the estuary (Neu 1960; Toodesh 2012). Future work could aim to account for a greater amount of variance in the biological data by incorporating information on current speeds, wave climate, sediment deposition and erosion rates, and salinity; these could be obtained from hydrodynamic model outputs (Warwick and Uncles 1980), or via direct field measurements.

Chemical variables were generally less important predictors of invertebrate community characteristics compared to the physical variables, although organic carbon content was the most important predictor of overall abundance. Organic carbon content is a key variable influencing infaunal abundance and composition (Ramey and Snelgrove 2003; Lutz-Collins and Quijón 2014), and excessive organic content can be detrimental (Gray 2002; Ellis et al. 2017). However, at TOC levels lower than 10 mg/g (1%), there is expected to be little risk of negative impacts on biodiversity (Hyland et al. 2005; Magni et al. 2009). Since most samples in the current study had TOC values within this range, it is not surprising that it was not among the more important predictors of species richness, Shannon diversity, or AMBI.

High metal concentrations have clear acute effects on invertebrate communities (Ward and Hutchings 1996; Fukunaga et al. 2010), but at low concentrations, chronic effects can still be detected (Nelson et al. 1988; Ellis et al. 2017). Although a previous study using samples from the same sites in Saint John Harbour did not find a link between contaminant levels and abundances of two potential indicator species (Pippy et al. 2016), the larger dataset used here showed that relationships between metal contamination and biological metrics were weak but detectable, even at levels considered environmentally ‘acceptable’. Samples with higher concentrations of certain metals (e.g. mercury) did have lower diversity and higher AMBI, even though these concentrations were still below thresholds of concern. It is worth noting that while lanthanum and/or phosphorus were identified as possibly important predictors in some models (particularly species richness and 7 of the best 10 DistLM models), these are a priori unlikely candidates to have strong negative influences at the recorded concentrations and were confounded with depth (La: Pearson’s *ρ* = −0.373, *p* < 0.001; P: *ρ* = −0.521, *p* < 0.001).

### Spatial and Temporal Trends in Saint John Harbour

Spatial trends were largely in line with expectations. Contaminant concentrations, TOC, and AMBI tended to be higher in the Inner Harbour, while diversity and richness were higher in the Outer Harbour. This was most likely related to depth, which was identified as the most important predictor for the biological metrics. Multivariate analysis identified more significant variation among sites than analyses of the univariate indices; this is one of the advantages of the multivariate approach (Gray et al. 1990; Ward and Hutchings 1996). Inner Harbour site 1 was particularly distinct from the other sites, reflecting lower overall abundance and taxonomic richness and the absence of several species – particularly bivalve molluscs – commonly recorded at the other sites. This may reflect hydrodynamic conditions, as discussed above. Outer Harbour site 6 also had a distinct community, with comparatively low abundances of several polychaete species, as well as the presence of several species such as the reef-building polychaete *Sabellaria vulgaris* and the amphipod *Unciola irrorata* which were absent or very rare at the other sites. These community differences were related to depth (site 6 was in deeper water than the other sites) and sediment grain size distribution (site 6 had a much lower proportion of silt and clay fractions than the other sites), rather than to contaminant concentrations, which did not differ from the other Outer Harbour sites.

Anthropogenic influences in Saint John Harbour have changed between 2011 and 2021. Among the most potentially important changes were improvements in municipal wastewater treatment (City of Saint John 2014); similar changes in domestic waste management in other estuaries have been associated with improvements in benthic habitats (Rutecki et al. 2020; Zeldis et al. 2020). Monitoring of benthic invertebrates can often detect effects resulting from changes in local or regional anthropogenic pressures (Dolbeth et al. 2007; Veríssimo et al. 2012; Wang et al. 2017; Umehara et al. 2022). However, while there were considerable interannual fluctuations in most of the invertebrate community metrics, there was little evidence for substantial background change in the environmental status of Saint John Harbour. This implies either that the reference sites were not sufficiently impacted by the release of untreated sewage in the harbour for effects of the changes in water quality to be detectable at these locations or that insufficient time has passed for changes to be observed, since recovery of soft-sediment macroinvertebrates from effects of wastewater discharge can take more than 10 years (Borja et al. 2006).

The clearest biological change occurred at Outer Harbour site 4, where abundance, richness, and species composition differed between the early years (2011–2013) and the later years (2017–2020). The increase in species richness was particularly large, from around 15 species in 2011–2013 to almost 30 species in 2020. It is not clear whether these changes indicate decline or improvement; increases in overall abundance and abundances of polychaete species such as *Cossura longocirrata* (an opportunist; Olsgard and Hasle 1993) imply environmental decline, while increasing richness and modest increases in abundances of some more sensitive taxa (e.g. *Terebellides stroemii*, *Nucula proxima*, *Ennucula delphinodonta*, *Ilyanassa trivittata*, *Eudorella truncatula*) suggest improvement. There was no corresponding shift in contamination or sediment conditions, apart from possibly the increase in lead concentrations. However, the change in measured lead concentrations was evident at all sites (particularly in the Inner Harbour) and remains unexplained. There were also some changes in the abundance of the polychaete *Micronephthys neotena*, which first appeared in reference site samples in 2017 and increased in number at sites 2 and 3 through 2019 and 2020. *Micronephthys* has been increasing in Boston Harbour since the 1990s, concurrent with reductions in sewage discharge and other improvements (Rutecki et al. 2022), suggesting that an increase in Saint John Harbour might be indicative of positive change. However, other sources suggest that *M. neotena* is an opportunistic species (Noyes 1980; Grenon 1981; Dnestrovskaya and Jirkov 2010), and in the Gulf of St Lawrence, *M. neotena* was recorded close to contaminant sources (Dreujou et al. 2020). This species should be monitored to see if increases from 2017 to 2020 are part of an ongoing trend.

### Setting a Baseline of ‘Normal’ Ranges

The ‘defining normal’ approach (Arciszewski and Munkittrick 2015) was originally developed for monitoring of fish health in freshwater systems and has not been applied in marine benthic systems. We adopted this approach to determine baseline biological, physical, and chemical values for the individual reference sites, for the Inner and Outer Harbour areas, and for Saint John Harbour overall. These ‘baseline’ data can be used to monitor for changes at the reference sites (resulting from local, regional, or global effects) and to determine whether sites closer to potential sources of contamination show evidence of localised anthropogenic impact.

AMBI scores place Outer Harbour sites within the ‘slightly polluted’ category (AMBI between 1.2 and 3.3; Borja et al. 2000), while the Inner Harbour sites straddle the ‘slightly polluted’ and ‘moderately polluted/transitional to pollution’ categories (AMBI up to 4.3). While these values may be considered somewhat elevated for ‘reference’ sites, this may reflect comparatively high levels of natural disturbance resulting from tidal influences, rather than effects of pollution, since some pollution-tolerant species may also be tolerant of natural stressors, leading to indices such as AMBI incorrectly classifying naturally stressed sites as polluted (Dauvin and Ruellet 2009). The relatively clear distinction between the AMBI scores in the Inner and Outer Harbour may therefore reflect differences in hydrodynamic stresses, which typically decline with increasing depth (Dutertre et al. 2013).

## Conclusions

Coastal sediments are repositories of contaminants released from human activities, and the abundance and diversity of infaunal communities can reflect the chemical and physical degradation of their habitat. However, to evaluate the extent and magnitude of impacts on these communities, it is critical to understand the factors that predict species composition and abundance, particularly within the range of normal conditions which can be expected in the absence of acute anthropogenic stressors. While this is reasonably well-understood for many coastal systems, few studies have examined regions that are highly affected by tidal fluxes. Measures of physical, chemical, and biological characteristics of sediments collected from reference areas in a highly tidal harbour showed that patterns in invertebrate diversity metrics and community composition were predominantly determined by naturally driven factors. Despite improved municipal wastewater treatment and other changes in human activities, over a 10-year period, there were few significant regional changes in contamination or invertebrate communities. Sediment contaminant concentrations were below most sediment quality guidelines, likely as a result of the highly dynamic nature of Saint John Harbour. These results support previous findings that benthic communities in naturally ‘stressed’ environments are comparatively resilient (Bolam et al. 2011; Callaway et al. 2020; Foulquier et al. 2020).

## Supplementary Information

Below is the link to the electronic supplementary material.

Supplementary file1 (DOCX 951 KB)

## Data Availability

Data are available from the corresponding author on request. Some of the data can be downloaded from the following link: https://catalogue.ogsl.ca/en/dataset/ca-cioos_1954d7f6-0fc1-42fa-b56c-0cae5de365ae.
