# Supplementary material for: Temporal and Spatial Trends in Benthic Infauna and Potential Drivers, in a Highly Tidal Estuary in Atlantic Canada
Source: Estuaries Coast. 2023 Jun 13;46(6):1612–31. doi: 10.1007/s12237-023-01222-w (PMC10371968; doi:10.1007/s12237-023-01222-w)

**Supplementary Material for**: Temporal and spatial trends in benthic infauna, and potential drivers, in a highly tidal estuary in Atlantic Canada.

Contents:

Supplementary Table S1: Additional information on physical and chemical analyses 2

Supplementary Table S2: Comparison of UNB and RPC metals data, 2018 samples 3

Supplementary Table S3: Normal ranges for physical, chemical, and biological variables 4

Supplementary Table S4: Two-way ANOVA global tests for univariate metrics 8

Supplementary Table S4: Pairwise comparisons for PERMANOVA analysis 9

Supplementary Table S5: Abundances of invertebrate species identified by BVSTEP 11

Supplementary Table S6: PCA summary for UNB metals data, 2011-2018 12

Supplementary Table S7: PCA summary for RPC metals data, 2018-2021 13

Supplementary Table S8: PLSR model summaries 14

Figure S1: Sample nMDS ordination 18

Figure S2: Temporal trends in abundances for BVSTEP-identified species at Site 1 19

Figure S3: Temporal trends in abundances for BVSTEP-identified species at Site 2 20

Figure S4: Temporal trends in abundances for BVSTEP-identified species at Site 3 21

Figure S5: Temporal trends in abundances for BVSTEP-identified species at Site 4 22

Figure S6: Temporal trends in abundances for BVSTEP-identified species at Site 13 23

Figure S7: Temporal trends in abundances for BVSTEP-identified species at Site 6 24

Figure S8: Mean TOC for each site on each date 25

Figure S9: Mean Total PAH concentration for each site on each date 26

Figure S10: Mean Total mercury concentration for each site on each date 27

Figure S11: Mean values for PC1-PC3 from PCA for UNB metals data (2011-2018) 28

Figure S12: Lanthanum and lead concentrations at reference sites, UNB data (2011-2018) 29

Figure S13: Chromium and lead concentrations at reference sites, RPC data (2018-2021) 30

Supplementary Table S1. Additional information on physical and chemical analyses

a) Summary of physical and chemical analyses of sediment samples.

| Test | Sample Mass | Units | Method |
| --- | --- | --- | --- |
| LOI550/950 | 20 g wet | % | Gravimetric |
| TOC | Calculated from LOI550 | % | Calculated from linear regression  % TOC = (0.255 x % LOI550) - 0. 111, R2 = 0.882 |
| Grain Size | 10-50 g dry | % | Gravimetric (Folk 1980) |
| Elemental (Metals) | 0.5 g dry | mg/kg dw | Digestion and ICP-OES quantification based on US EPA 3051, 200.7, and 6010C methods |
| Total Hg | 0.03 g dry | µg/kg dw | DMA-80 based on US EPA 7473 methods |
| PAHs | ≥10 g dry | mg/kg dw | ASE extraction, GPC cleanup and GC/MS quantification based on US EPA 3545, 3640A and 8270C methods |

b) List of elements analysed by UNB (2011-2018) and RPC (2018-2021)

| Element | Symbol | UNB | RPC |
| --- | --- | --- | --- |
| Aluminium | Al | Y | Y |
| Arsenic | As | Y | Y |
| Boron | B |  | Y |
| Barium | Ba |  | Y |
| Calcium | Ca |  | Y |
| Cobalt | Co | Y | Y |
| Chromium | Cr | Y | Y |
| Copper | Cu | Y | Y |
| Iron | Fe | Y | Y |
| Potassium | K |  | Y |
| Lithium | Li |  | Y |
| Lanthanum | La | Y |  |
| Magnesium | Mg | Y | Y |
| Manganese | Mn | Y | Y |
| Sodium | Na |  | Y |
| Nickel | Ni | Y | Y |
| Phosphorous | P | Y |  |
| Lead | Pb | Y | Y |
| Rubidium | Rb | Y | Y |
| Sulphur | S | Y |  |
| Strontium | Sr | Y | Y |
| Vanadium | V | Y | Y |
| Zinc | Zn | Y | Y |

c) List of PAHs quantified, and quantification ions used.

| PAH | Quantification Ion |
| --- | --- |
| Acenaphthene | 153 |
| Acenaphthylene | 152 |
| Anthracene | 178 |
| Benzo[a]anthracene | 228 |
| Benzo[a]pyrene | 252 |
| Benzo[b]fluoranthene | 252 |
| Benzo[k]fluoranthene | 252 |
| Benzo[g,h,i]perylene | 276 |
| Dibenzo[a,h]anthracene | 278 |
| Chrysene | 228 |
| Fluoranthene | 202 |
| Fluorene | 166 |
| Indeno[1,2,3-cd]pyrene | 276 |
| Naphthalene | 128 |
| Phenanthrene | 178 |
| Pyrene | 202 |

Supplementary Table S2. Comparison of UNB and RPC laboratory trace element measurements for the same set of 65 samples collected in 2018. Means were compared using paired 1-way t-tests, variances were compared using the Levene test, and relationships between the two sets of measurements were examined by linear regression. Bold highlight indicates cases where differences were significant (*p* < 0.05), grey shaded cells indicate lack of significant difference.

For all elements except copper and nickel, UNB measurements were significantly higher, and some differences were large. Variance was also significantly higher for UNB measurements, except for copper, nickel, iron, lead and zinc. Relationships between UNB and RPC measurements on individual samples were statistically significant for all elements, but r^2^ values were typically low, indicating weak relationships with substantial noise. Furthermore, application of a regression-based correction to the full dataset would have required extrapolation beyond the range of values measured in 2018 for some elements.

As there were no significant differences in mean or variance for copper or nickel, data from the two laboratories for these metals could be considered comparable without adjustment. Two further metals, lead and zinc, are candidates for mathematical correction; variances were comparable between the two laboratories and measurements were strongly correlated (r^2^ >= 0.89). Nevertheless, since this was not the case for most metals, mathematical correction of RPC data (to align with UNB data) was generally not appropriate; data from the two laboratories were analysed separately.

| Element | UNB | | RPC | | Paired t (1-tailed) | | Levene test | | Regression | | Notes |
| --- | --- | --- | --- | --- | --- | --- | --- | --- | --- | --- | --- |
|  | Mean | SD | Mean | SD | t | *p* | F | *p* | r^2^ | *p* |  |
| Silver |  |  |  |  |  |  |  |  |  |  | Almost all measurements < DL |
| Aluminium | 20644.62 | 5607.89 | 11381.23 | 2212.94 | **-15.792** | **< 0.001** | **26.161** | **< 0.001** | **0.301** | **< 0.001** |  |
| Arsenic | 7.56 | 1.92 | 5.15 | 1.21 | **-12.269** | **< 0.001** | **6.541** | **0.012** | **0.364** | **< 0.001** |  |
| Cadmium |  |  |  |  |  |  |  |  |  |  | All UNB measurements < DL |
| Cobalt | 10.38 | 1.83 | 8.74 | 1.34 | **-7.693** | **< 0.001** | **4.193** | **0.043** | **0.209** | **< 0.001** |  |
| Chromium | 25.6 | 6.26 | 19.51 | 3.74 | **-9.593** | **< 0.001** | **8.913** | **0.003** | **0.334** | **< 0.001** |  |
| Copper | 11.81 | 7.21 | 12.17 | 6.25 | 0.99 | 0.837 | 0.943 | 0.677 | **0.89** | **< 0.001** | Correction not required |
| Iron | 21723.08 | 4791.26 | 20244.62 | 3576.23 | **-2.469** | **0.008** | 2.91 | 0.09 | **0.129** | **0.003** |  |
| Magnesium | 6457.85 | 1580 | 6459.54 | 1163.42 | **-2.174** | **0.017** | **4.903** | **0.029** | **0.208** | **< 0.001** |  |
| Manganese | 469.51 | 121.45 | 373.49 | 75.68 | **-7.33** | **< 0.001** | **6.917** | **0.01** | **0.3** | **< 0.001** |  |
| Nickel | 19.76 | 3.91 | 19.08 | 3.30 | -1.577 | 0.06 | 1.158 | 0.284 | **0.298** | **< 0.001** | Correction not required |
| Lead | 16.89 | 6.54 | 13.57 | 6.66 | **-8.747** | **< 0.001** | 0.059 | 0.809 | **0.898** | **< 0.001** | Simple correction possible |
| Rubidium | 31.90 | 11.31 | 13.89 | 4.00 | **-14.904** | **< 0.001** | **44.366** | **< 0.001** | **0.47** | **< 0.001** |  |
| Selenium |  |  |  |  |  |  |  |  |  |  | All measurements < DL |
| Strontium | 51.16 | 12.66 | 36.02 | 8.37 | **-12.341** | **< 0.001** | **5.386** | **0.022** | **0.425** | **< 0.001** |  |
| Thallium |  |  |  |  |  |  |  |  |  |  | All UNB measurements < DL |
| Uranium |  |  |  |  |  |  |  |  |  |  | Most UNB measurements < DL |
| Vanadium | 40.82 | 8.80 | 29.35 | 4.82 | **-11.91** | **< 0.001** | **10.29** | **< 0.001** | **0.229** | **< 0.001** |  |
| Zinc | 61 | 40.06 | 57.66 | 31.78 | **-1.835** | **0.036** | 0.242 | .0624 | **0.893** | **< 0.001** | Simple correction possible |

Supplementary Table S3. Normal ranges for invertebrate community metrics, physical and chemical variables, based on the 6 reference sites. Calculations use the mean values for each sampling date at each site as replicates, normal ranges are ±2SD above and below the relevant mean. Green shaded cells indicate data used for calculation of Inner Harbour normal ranges, blue shaded cells indicate data used for calculation of Outer Harbour normal ranges. Where sites are unshaded, this indicates that data were not used for calculation of normal ranges for Inner Harbour, Outer Harbour, or Saint John Harbour overall.

a) Normal ranges for univariate invertebrate community metrics. Since significant changes over time were observed in abundance (at Sites 1, 4, 13) and species richness (at Sites 4 and 13), separate normal ranges were calculated for 2011-2013 and 2017-2020 for these sites, and for Outer Harbour and Overall Saint John Harbour normal ranges for abundance and richness. Site 1 was not included in calculation of Inner Harbour Combined normal ranges for abundance and species richness, since it differed substantially from Sites 2 and 3. Site 6 was not included in calculation of the Outer Harbour Combined normal range for Shannon Diversity, since it differed from Sites 4 and 13. 2011 data were not used for calculation of abundance ranges for Sites 2 and 3, as values were atypically high. 2019 Site 6 data were not used for calculation of abundance or Shannon diversity values, since data were atypical for that year (a slightly different location was sampled). Information on AMBI condition categories is shown below (adapted from Borja et al 2000).

|  |  | INNER HARBOUR | | | | OUTER HARBOUR | | | | OVERALL SJH |
| --- | --- | --- | --- | --- | --- | --- | --- | --- | --- | --- |
|  |  | Site 1 | Site2 | Site 3 | Combined | Site 4 | Site 13 | Site 6 | Combined |  |
| Abundance (ind.m^-2^) | 2011-2013 | 1886 - 3457 |  |  |  | 2804 - 5740 | 1447 - 7479 |  | 1005 - 6883 | 0 - 13,739 |
| (ind per grab / 0.032) | 2017-2020 | 528 - 883 |  |  |  | 8515 - 11862 | 5003 - 7711 |  | 647 - 12,985 | 0 - 16,108 |
|  | Full |  | 2801 -16,353 | 7999 - 17,049 | 4735 - 17,348 |  |  | 410 - 5620 |  |  |
| Species Richness | 2011-2013 |  |  |  |  | 15 - 18 | 10 - 23 |  | 10 - 26 | 8 - 25 |
|  | 2017-2020 |  |  |  |  | 20 - 30 | 18 - 26 |  | 17 - 29 | 5 - 32 |
|  | Full | 3 - 12 | 12 - 22 | 15 - 22 | 13 - 22 |  |  | 14 - 29 |  |  |
| Shannon Diversity | Full | 0.89 - 1.85 | 0.59 - 2.50 | 0.82 - 2.08 | 0.76 - 2.15 | 1.74 - 2.28 | 1.41 - 2.02 | 1.73 - 3.02 | 1.45 - 2.27 | 0.85 - 2.61 |
| AMBI | Full | 1.87 - 4.69 | 1.90 - 4.39 | 2.49 - 4.17 | 2.13 - 4.38 | 1.34 - 2.86 | 0.46 - 1.86 | 0.72 - 1.86 | 0.44 - 2.59 | 0.30 - 4.43 |

| AMBI | INFAUNAL STATUS | SITE STATUS |
| --- | --- | --- |
| ≤ 0.2 | Normal | Undisturbed |
| 0.2 - 1.2 | Impoverished | Undisturbed |
| 1.2 - 3.3 | Unbalannced | Slightly disturbed |
| 3.3 - 4.3 | Transitional to disturbance | Disturbed |
| 4.3 - 5 | Disturbed | Disturbed |
| 5 - 5.5 | Transitional to heavy disturbance | Heavily disturbed |
| 5.5 - 6 | Heavily disturbed | Heavily disturbed |
| 7 | Azoic | Extremely disturbed |

b) Normal ranges for mercury (Hg; n=10 for sites, n=30 for Inner Harbour Combined, n=29 for Outer Harbour combined, n=59 for Overall Saint John Harbour), total organic carbon (TOC; n=11 for sites, n=33 for Inner Harbour Combined, n=32 for Outer Harbour combined, n=65 for Overall Saint John Harbour) and total polycyclic aromatic hydrocarbons (TPAH; n=8 for sites, n=24 for Inner Harbour Combined, n=23 for Outer Harbour combined, n=47 for Overall Saint John Harbour). For all variables n is reduced by 1 for Site 13, which was not sampled on the first date.

|  | INNER HARBOUR | | | | OUTER HARBOUR | | | | OVERALL SJH | MAX |
| --- | --- | --- | --- | --- | --- | --- | --- | --- | --- | --- |
|  | Site 1 | Site 2 | Site 3 | Combined | Site 4 | Site 13 | Site 6 | Combined |  |  |
| Hg | 2.33 - 21.7 | 7.39 - 13.4 | 6.69 - 15.1 | 4.83 - 17.4 | 4.4 - 8.82 | 5.89 - 12.3 | 3.81 - 9.48 | 3.85 - 11 | 2.98 - 15.6 | 41 |
| TOC | 0.131 - 1.37 | 0.383 - 1.39 | 0.463 - 1.29 | 0.319 - 1.36 | 0.264 - 0.994 | 0.374 - 0.987 | 0.368 - 0.851 | 0.334 - 0.943 | 0.27 - 1.21 | 1.71 |
| TPAH | 0.003 - 0.33 | 0.005 - 0.36 | 0.024 - 0.311 | 0.017 - 0.328 | 0 - 0.297 | 0.047 - 0.366 | 0 - 0.326 | 0 - 0.334 | 0.003 - 0.33 | 3.13 |

c) Normal ranges for grain size categories. Range calculations based on mean values from all dates (2011-2021) excluding April 2012. Sites, n=10 for all sites except for Site 13 (n =9); Inner Harbour Combined, n = 30; Outer Harbour Combined, n = 19 (‘Outer Harbour Combined’ for these measures does not include Site 6, which was distinct from all other sites). An ‘Overall Saint John Harbour’ estimate is not provided, as sites varied enough that a harbour-wide average range was of little value

|  | INNER HARBOUR | | | | OUTER HARBOUR | | | |
| --- | --- | --- | --- | --- | --- | --- | --- | --- |
|  | Site 1 | Site 2 | Site 3 | Combined | Site 4 | Site 13 | Site 6 | Combined |
| % Silt and Clay | 64.99 - 100 | 56.71 - 100 | 59.53 - 100 | 60.97 - 100 | 62.55 - 93.87 | 53.72 - 88.62 | 26.7 - 70.38 | 57.27 - 92.49 |
| % Fine and Medium Sand | 0 - 24.35 | 0 - 40.15 | 0 - 36.69 | 0 - 33.99 | 6.31 - 34.49 | 11.61 - 40.83 | 22.83 - 58.5 | 8.00 - 38.31 |
| % Coarse Sand | 0 - 11.01 | 0 - 3.31 | 0 - 3.85 | 0 - 7.02 | 0 - 2.99 | 0 - 5.9 | 0 - 12.07 | 0 - 4.57 |
| % Granules and Pebbles | 0 - 2.4 | 0 - 0.64 | 0 - 0.88 | 0 - 1.52 | 0 - 0.7 | 0 - 2.69 | 0 - 22.66 | 0 - 1.92 |

d) Normal ranges for elements measured by UNB (2011-2018) and RPC (2018-2021). UNB: site ranges, n=10 (except for Site 13, n=9); Inner Harbour Combined ranges, n=30; Outer Harbour Combined ranges, n=29; Overall SJH ranges, n=59. RPC: site ranges, n=4; Inner / Outer Harbour Combined ranges, n=12; Overall SJH range, n=24.

|  |  | INNER HARBOUR | | | | OUTER HARBOUR | | | | OVERALL  SJH | MIN | MAX |
| --- | --- | --- | --- | --- | --- | --- | --- | --- | --- | --- | --- | --- |
|  |  | Site 1 | Site 2 | Site 3 | Combined | Site 4 | Site 13 | Site 6 | Combined |  |  |  |
| Ag | UNB | NA | NA | NA | NA | NA | NA | NA | NA | NA | < DL | 0.61 |
|  | RPC | NA | NA | NA | NA | NA | NA | NA | NA | NA | < DL | < DL |
| Al | UNB | 9530 - 26400 | 11600 - 33000 | 13800 - 26000 | 11000 - 29100 | 10000 - 24100 | 11700 - 24500 | 12100 - 21400 | 11300 - 23300 | 10600 - 26800 | 8420 | 40200 |
|  | RPC | 4240 - 17300 | 9010 - 16000 | 9050 - 14000 | 7250 - 15900 | 5540 - 13900 | 8310 - 11900 | 6260 - 13300 | 6840 - 12900 | 6670 - 14800 | 5490 | 18000 |
| As | UNB | 4.2 - 9.99 | 4.78 - 10.1 | 5.73 - 8.6 | 4.88 - 9.57 | 4.56 - 7.28 | 4.53 - 8.32 | 4.47 - 8.17 | 4.51 - 7.92 | 4.45 - 9.01 | 3.87 | 14.92 |
|  | RPC | 3.05 - 8.55 | 4.01 - 7.79 | 3.9 - 7.3 | 3.79 - 7.74 | 3.53 - 5.17 | 3.47 - 5.13 | 3.08 - 7.02 | 3.17 - 5.96 | 3.09 - 7.24 | 2 | 11 |
| B | RPC | 2.93 - 34.5 | 14.7 - 27.7 | 15.7 - 23.8 | 10.5 - 29.3 | 8.48 - 22.2 | 14.1 - 17.3 | 10.7 - 19.4 | 11 - 19.7 | 9.1 - 26.2 | 7 | 40 |
| Ba | RPC | 56.5 - 77.5 | 61 - 71.9 | 55.7 - 87.4 | 57 - 79.7 | 58.7 - 99.5 | 59.5 - 94.2 | 41.3 - 80.6 | 48.1 - 96.5 | 51.4 - 89.2 | 37 | 113 |
| Be | RPC | 0.219 - 0.841 | 0.49 - 0.83 | 0.473 - 0.737 | 0.372 - 0.825 | 0.306 - 0.734 | 0.463 - 0.577 | 0.315 - 0.675 | 0.361 - 0.663 | 0.347 - 0.763 | 0.3 | 1 |
| Ca | RPC | 7370 - 11700 | 7590 - 11200 | 8040 - 9860 | 7660 - 10900 | 7130 - 9770 | 7820 - 8850 | 6430 - 11700 | 6920 - 10300 | 7190 - 10700 | 5750 | 13300 |
| Cd | UNB | NA | NA | NA | NA | NA | NA | NA | NA | NA | < DL | 0.21 |
|  | RPC | 0.034 - 0.067 | 0.031 - 0.051 | 0.031 - 0.054 | 0.030 - 0.060 | 0.0278- 0.048 | 0.026 - 0.045 | 0.034 - 0.054 | 0.027 - 0.051 | 0.027 - 0.056 | 0.02 | 0.1 |
| Co | UNB | 5.85 - 11.6 | 6.1 - 13.3 | 7.71 - 11.2 | 6.42 - 12.2 | 6.43 - 9.89 | 6.67 - 11.5 | 7.82 - 9.81 | 6.78 - 10.6 | 6.49 - 11.5 | 5.34 | 15.96 |
|  | RPC | 3.28 - 12.7 | 7.29 - 11.4 | 7.31 - 9.93 | 5.65 - 11.7 | 4.84 - 10.2 | 6.94 - 8.76 | 5.6 - 10.9 | 5.75 - 9.98 | 5.59 - 10.9 | 4.5 | 14.5 |
| Cr | UNB | 15.4 - 32.5 | 19.3 - 37.2 | 20.8 - 33.1 | 17.9 - 34.9 | 17.8 - 26.7 | 17.3 - 32.4 | 17.8 - 26.6 | 17.1 - 29 | 16.7 - 32.8 | 13.4 | 42.9 |
|  | RPC | 7.58 - 29.4 | 16.2 - 26.1 | 16.2 - 22.8 | 12.8 - 26.6 | 9.48 - 23.2 | 14.3 - 19 | 11 - 22.1 | 11.7 - 21.3 | 11.5 - 24.8 | 10 | 31 |
| Cu | UNB | 4.28 - 11.8 | 4.77 - 12.1 | 4.22 - 11.4 | 4.52 - 11.7 | 0.47 - 10.9 | 1.21 - 13.2 | 1.67 - 9.55 | 1.02 - 11.2 | 2.34 - 11.9 | < DL | 17.12 |
|  | RPC | 3.47 - 15.6 | 7.56 - 13 | 7.82 - 11.5 | 6.16 - 13.5 | 4.25 - 10.9 | 5.94 - 9.76 | 5.61 - 9.99 | 5.42 - 10 | 5.09 - 12.5 | 4 | 18 |
| Fe | UNB | 14900 - 28600 | 15800 - 32400 | 17600 - 27300 | 15900 - 29600 | 13300 - 26300 | 18000 - 24200 | 16500 - 25200 | 15700 - 25400 | 15400 - 28000 | 13100 | 43000 |
|  | RPC | 9040 - 29500 | 16300 - 27500 | 16500 - 24400 | 13700 - 27400 | 11300 - 23500 | 16200 - 20500 | 12100 - 24700 | 13200 - 22900 | 13000 - 25600 | 10800 | 33100 |
| K | RPC | 296 - 4530 | 1910 - 4120 | 1960 - 3530 | 1310 - 4130 | 1010 - 3400 | 1770 - 2810 | 1120 - 3110 | 1330 - 3070 | 1200 - 3730 | 1070 | 4670 |
| La | UNB | 14.3 - 27.8 | 15.1 - 27.4 | 14.3 - 27 | 14.7 - 27.2 | 16.4 - 22.5 | 14.8 - 23.7 | 13.2 - 21.7 | 14.5 - 23 | 14.1 - 25.6 | 10.8 | 37.6 |
| Li | RPC | 9.38 - 36.3 | 19.9 - 33.7 | 19.5 - 29.3 | 15.7 - 33.7 | 12.5 - 28.3 | 18 - 24 | 14.2 - 27.9 | 15.1 - 26.5 | 14.4 - 31.1 | 11.4 | 38.5 |
| Mg | UNB | 4050 - 8400 | 4470 - 9480 | 4990 - 8540 | 4470 - 8850 | 4470 - 6610 | 5090 - 7140 | 4540 - 7360 | 4620 - 7100 | 4320 - 8210 | 3520 | 11600 |
|  | RPC | 2610 - 10300 | 4920 - 9190 | 5020 - 8060 | 4190 - 9190 | 3340 - 7720 | 4920 - 6570 | 4130 - 7560 | 4160 - 7250 | 3930 - 8460 | 3300 | 11100 |
| Mn | UNB | 286 - 661 | 311 - 647 | 333 - 601 | 314 - 633 | 320 - 482 | 307 - 473 | 322 - 424 | 314 - 462 | 280 - 582 | 282 | 902 |
|  | RPC | 127 - 757 | 282 - 542 | 323 - 462 | 229 - 602 | 217 - 421 | 265 - 421 | 227 - 444 | 242 - 422 | 207 - 540 | 231 | 908 |
| Mo | RPC | 0.202 - 0.628 | 0.178 - 0.642 | 0.19 - 0.52 | 0.199 - 0.587 | 0.19 - 0.43 | 0.182 - 0.418 | 0.23 - 0.57 | 0.181 - 0.493 | 0.183 - 0.547 | 0.2 | 0.8 |
| Na | RPC | 0 - 17000 | 5150 - 13100 | 5860 - 11100 | 3370 - 13900 | 2230 - 10000 | 4510 - 7400 | 3590 - 7020 | 3330 - 8250 | 2260 - 12200 | 2580 | 21000 |

d) continued

|  |  | INNER HARBOUR | | | | OUTER HARBOUR | | | | OVERALL  SJH | MIN | MAX |
| --- | --- | --- | --- | --- | --- | --- | --- | --- | --- | --- | --- | --- |
|  |  | Site 1 | Site 2 | Site 3 | Combined | Site 4 | Site 13 | Site 6 | Combined |  |  |  |
| Ni | UNB | 10.6 - 23.6 | 13.3 - 25 | 14.8 - 22.3 | 12.7 - 23.8 | 11.1 - 19.3 | 11.5 - 23.3 | 14 - 18.7 | 11.8 - 20.8 | 11.9 - 22.7 | 7.9 | 31.3 |
|  | RPC | 7.28 - 27 | 15.8 - 24.1 | 15.5 - 20.9 | 12.2 - 24.7 | 9.49 - 21 | 14 - 17.6 | 11.2 - 21.5 | 11.5 - 20.1 | 11.2 - 23 | 9 | 31 |
| P | UNB | 476 - 859 | 523 - 796 | 470 - 842 | 494 - 828 | 413 - 643 | 359 - 662 | 246 - 792 | 333 - 706 | 365 - 818 | 310 | 2123 |
| Pb | UNB | 5.49 - 17 | 3.54 - 21.6 | 5.06 - 19.3 | 4.74 - 19.3 | 4.72 - 16.3 | 5.56 - 19.6 | 4.78 - 15 | 4.73 - 17.2 | 4.69 - 18.3 | 4.87 | 24.1 |
|  | RPC | 3.92 - 17.2 | 8.62 - 15.4 | 9.23 - 13.2 | 7.04 - 15.5 | 6.03 - 12.8 | 8.65 - 12.4 | 7.23 - 11.3 | 7.17 - 12.3 | 6.74 - 14.2 | 5.1 | 19.4 |
| Rb | UNB | 7.98 - 67.7 | 21.4 - 82.4 | 18.2 - 79.7 | 14.4 - 78 | 15.5 - 58.5 | 5.53 - 81.9 | 11.2 - 60.8 | 10.3 - 67.2 | 11.7 - 73.4 | 3.98 | 106.5 |
|  | RPC | 3.43 - 23.2 | 11.4 - 22 | 11.2 - 18.7 | 8.17 - 21.8 | 5.81 - 18.2 | 9.88 - 14.9 | 6.08 - 17.6 | 7.44 - 16.7 | 7.12 - 20 | 5.6 | 24.4 |
| S | UNB | 870 - 2350 | 1140 - 2470 | 1240 - 2360 | 1080 - 2400 | 907 - 1710 | 1230 - 2620 | 1360 - 2780 | 859 - 2660 | 967 - 2530 | 574 | 4870 |
| Sb | RPC | NA | NA | NA | NA | NA | NA | NA | NA | NA | < DL | 0.2 |
| Se | UNB | NA | NA | NA | NA | NA | NA | NA | NA | NA | < DL | 3.25 |
|  | RPC | NA | NA | NA | NA | NA | NA | NA | NA | NA | < DL | < DL |
| Sn | RPC | NA | NA | NA | NA | NA | NA | NA | NA | NA | < DL | 0.93 |
| Sr | UNB | 30.4 - 66 | 35.9 - 71.9 | 43.7 - 58.9 | 35.7 - 66.6 | 35 - 51.4 | 33.4 - 53.7 | 33.7 - 59.3 | 33.8 - 55.1 | 33 - 62.7 | 25.6 | 103.5 |
|  | RPC | 20 - 59.8 | 31.7 - 53.6 | 33.5 - 45.2 | 28 - 53.2 | 20.2 - 45 | 27 - 35.6 | 26 - 41.4 | 24.3 - 40.7 | 23.3 - 49.9 | 19 | 70 |
| Tl | UNB | NA | NA | NA | NA | NA | NA | NA | NA | NA | < DL | 0.88 |
|  | RPC | NA | NA | NA | NA | NA | NA | NA | NA | NA | < DL | 0.4 |
| U | UNB | NA | NA | NA | NA | NA | NA | NA | NA | NA | < DL | 19.94 |
|  | RPC | 0.44 - 0.9 | 0.63 - 0.86 | 0.622 - 0.858 | 0.554 - 0.882 | 0.517 - 0.843 | 0.594 - 0.846 | 0.431 - 0.969 | 0.519 - 0.881 | 0.539 - 0.879 | 0.4 | 1.4 |
| V | UNB | 25.3 - 51.4 | 31.1 - 57.9 | 36.2 - 47.4 | 29.5 - 53.6 | 26 - 46.8 | 27.4 - 52.2 | 30.1 - 43 | 27.4 - 47.6 | 27.8 - 51.3 | 21.3 | 67.6 |
|  | RPC | 11.2 - 44.4 | 24.3 - 40.2 | 24.5 - 35.2 | 19.2 - 40.7 | 15.8 - 35.6 | 23.2 - 30.1 | 19.5 - 32.4 | 19.6 - 32.6 | 18.5 - 37.6 | 15 | 47 |
| Zn | UNB | 26.6 - 62.3 | 30.5 - 65.3 | 36.8 - 58.3 | 31.2 - 62.1 | 28.3 - 50.2 | 28.9 - 60.6 | 32.5 - 52.1 | 29.3 - 54.7 | 29.6 - 59.1 | 20.2 | 89.4 |
|  | RPC | 22.1 - 66.6 | 39.2 - 60.7 | 38.7 - 53.8 | 32.5 - 61.2 | 25.8 - 53.7 | 35.3 - 45.4 | 31.4 - 51.8 | 31 - 50.1 | 30.2 - 57.2 | 23 | 73 |

Supplementary Table S4. Two-way ANOVA global tests for univariate metrics with Site and Year as fixed factors, and with Levene’s test to compare variance among groups.

1. Levene’s test of variance among levels

| Variable | df | F | *p* |
| --- | --- | --- | --- |
| Abundance | 35,151 | 1.431 | 0.073 |
| Species richness | 41,177 | 0.572 | 0.982 |
| Shannon diversity | 41,177 | 0.857 | 0.714 |
| AMBI | 34,147 | 0.951 | 0.552 |

1. ANOVA table - Abundance (square-root transformed)

|  | df | SS | MS | *F* | *p* |
| --- | --- | --- | --- | --- | --- |
| Site | 5 | 114210 | 22842.1 | 47.4111 | < 0.0001 |
| Year | 5 | 1533 | 906.5 | 1.8816 | 0.1007 |
| Site * Year | 25 | 56111 | 2244.5 | 4.6586 | < 0.0001 |
| Residual | 151 | 72750 | 481.8 |  |  |

1. ANOVA table - Species Richness

|  | df | SS | MS | *F* | *p* |
| --- | --- | --- | --- | --- | --- |
| Site | 5 | 4659.9 | 931.99 | 49.252 | < 0.0001 |
| Year | 6 | 497.7 | 82.96 | 4.384 | 0.0004 |
| Site * Year | 30 | 1778.0 | 59.27 | 3.132 | < 0.0001 |
| Residual | 177 | 3349.3 | 18.92 |  |  |

1. ANOVA table - Shannon Diversity

|  | df | SS | MS | *F* | *p* |
| --- | --- | --- | --- | --- | --- |
| Site | 5 | 21.9408 | 4.3882 | 42.0575 | < 0.0001 |
| Year | 6 | 4.8059 | 0.8010 | 7.6768 | < 0.0001 |
| Site * Year | 30 | 16.4608 | 0.5487 | 5.2589 | < 0.0001 |
| Residual | 177 | 18.4677 | 0.1043 |  |  |

1. ANOVA table - AMBI (Site 1 is not included)

|  | df | SS | MS | *F* | *p* |
| --- | --- | --- | --- | --- | --- |
| Site | 4 | 148.997 | 37.249 | 174.0329 | < 0.0001 |
| Year | 6 | 11.955 | 1.993 | 9.3094 | < 0.0001 |
| Site * Year | 24 | 15.694 | 0.654 | 3.0552 | < 0.0001 |
| Residual | 147 | 31.463 | 0.241 |  |  |

Supplementary Table S5. Pairwise PERMANOVA comparisons among Sites within Years (left columns) and among Years at Sites (right columns). Adjusted *p*-values calculated using the Benjamini and Hochberg (1995) method to account for multiple comparisons. Bold highlight indicates *p* < 0.05.

| Year | Sites | *t* | *p* (perm) | *p* (adj) | Perms |  | Site | Years | *t* | *p* (perm) | *p* (adj) | Perms |
| --- | --- | --- | --- | --- | --- | --- | --- | --- | --- | --- | --- | --- |
| 2011 | 1,2 | 3.04 | **0.0093** | **0.014** | 126 |  | 1 | 2011,2012 | 1.72 | **0.0063** | **0.014** | 792 |
|  | 1,3 | 2.57 | **0.0083** | **0.014** | 126 |  |  | 2011,2013 | 1.35 | 0.1182 | 0.13 | 126 |
|  | 1,4 | 2.77 | **0.0025** | **0.014** | 462 |  |  | 2011,2017 | 1.75 | **0.024** | **0.03** | 126 |
|  | 1,6 | 3.12 | **0.0027** | **0.014** | 462 |  |  | 2011,2018 | 1.39 | 0.0545 | 0.064 | 126 |
|  | 1,13 | 2.21 | **0.0065** | **0.014** | 126 |  |  | 2011,2019 | 1.48 | **0.0166** | **0.022** | 126 |
|  | 2,3 | 1.39 | **0.0315** | **0.039** | 126 |  |  | 2011,2020 | 2.04 | **0.0084** | **0.014** | 126 |
|  | 2,4 | 2.11 | **0.0024** | **0.014** | 462 |  |  | 2012,2013 | 1.50 | **0.0405** | **0.049** | 792 |
|  | 2,6 | 2.75 | **0.0027** | **0.014** | 462 |  |  | 2012,2017 | 1.90 | **0.002** | **0.014** | 792 |
|  | 2,13 | 1.81 | **0.008** | **0.014** | 126 |  |  | 2012,2018 | 1.71 | **0.0011** | **0.014** | 792 |
|  | 3,4 | 2.17 | **0.0026** | **0.014** | 462 |  |  | 2012,2019 | 1.73 | **0.0024** | **0.014** | 792 |
|  | 3,6 | 2.69 | **0.0021** | **0.014** | 461 |  |  | 2012,2020 | 2.51 | **0.0009** | **0.014** | 792 |
|  | 3,13 | 1.88 | **0.0085** | **0.014** | 126 |  |  | 2013,2017 | 1.10 | 0.3185 | 0.331 | 126 |
|  | 4,6 | 2.50 | **0.0023** | **0.014** | 462 |  |  | 2013,2018 | 0.95 | 0.5677 | 0.575 | 126 |
|  | 4,13 | 1.41 | **0.0098** | **0.015** | 462 |  |  | 2013,2019 | 1.22 | 0.1477 | 0.161 | 126 |
|  | 6,13 | 1.71 | **0.0019** | **0.014** | 462 |  |  | 2013,2020 | 2.31 | **0.0088** | **0.014** | 126 |
| 2012 | 1,2 | 2.69 | **0.0001** | **0.014** | 5022 |  |  | 2017,2018 | 0.92 | 0.6565 | 0.662 | 126 |
|  | 1,3 | 2.33 | **0.0011** | **0.014** | 792 |  |  | 2017,2019 | 1.22 | 0.1026 | 0.115 | 126 |
|  | 1,4 | 2.64 | **0.0009** | **0.014** | 792 |  |  | 2017,2020 | 1.82 | **0.0069** | **0.014** | 126 |
|  | 1,6 | 3.14 | **0.0004** | **0.014** | 1710 |  |  | 2018,2019 | 0.81 | 0.7859 | 0.789 | 126 |
|  | 1,13 | 2.63 | **0.0019** | **0.014** | 792 |  |  | 2018,2020 | 1.88 | **0.0075** | **0.014** | 126 |
|  | 2,3 | 1.06 | 0.3635 | 0.377 | 1284 |  |  | 2019,2020 | 1.76 | **0.0077** | **0.014** | 126 |
|  | 2,4 | 2.73 | **0.0011** | **0.014** | 1286 |  | 2 | 2011,2012 | 2.16 | **0.001** | **0.014** | 1285 |
|  | 2,6 | 3.45 | **0.0003** | **0.014** | 5029 |  |  | 2011,2013 | 2.11 | **0.0083** | **0.014** | 126 |
|  | 2,13 | 2.44 | **0.001** | **0.014** | 1279 |  |  | 2011,2017 | 1.85 | **0.0085** | **0.014** | 126 |
|  | 3,4 | 2.19 | **0.0066** | **0.014** | 126 |  |  | 2011,2018 | 1.86 | **0.0074** | **0.014** | 126 |
|  | 3,6 | 2.83 | **0.0013** | **0.014** | 792 |  |  | 2011,2019 | 1.64 | **0.0073** | **0.014** | 126 |
|  | 3,13 | 2.14 | **0.0082** | **0.014** | 126 |  |  | 2011,2020 | 2.98 | **0.0067** | **0.014** | 126 |
|  | 4,6 | 2.52 | **0.0012** | **0.014** | 792 |  |  | 2012,2013 | 2.28 | **0.0014** | **0.014** | 1286 |
|  | 4,13 | 1.61 | **0.008** | **0.014** | 126 |  |  | 2012,2017 | 1.45 | **0.0447** | 0.053 | 1286 |
|  | 6,13 | 1.92 | **0.0015** | **0.014** | 792 |  |  | 2012,2018 | 1.64 | **0.0012** | **0.014** | 1287 |
| 2013 | 1,2 | 3.29 | **0.0099** | **0.015** | 126 |  |  | 2012,2019 | 1.57 | **0.0125** | **0.017** | 1285 |
|  | 1,3 | 2.51 | **0.0155** | **0.021** | 126 |  |  | 2012,2020 | 2.36 | **0.0006** | **0.014** | 1285 |
|  | 1,4 | 2.60 | **0.0071** | **0.014** | 126 |  |  | 2013,2017 | 1.61 | **0.0081** | **0.014** | 126 |
|  | 1,6 | 2.96 | **0.0071** | **0.014** | 126 |  |  | 2013,2018 | 1.47 | **0.0163** | **0.022** | 126 |
|  | 1,13 | 2.72 | **0.0069** | **0.014** | 126 |  |  | 2013,2019 | 1.73 | **0.0084** | **0.014** | 126 |
|  | 2,3 | 1.62 | **0.0089** | **0.014** | 126 |  |  | 2013,2020 | 3.25 | **0.0091** | **0.014** | 126 |
|  | 2,4 | 2.14 | **0.0071** | **0.014** | 126 |  |  | 2017,2018 | 0.59 | 0.9607 | 0.961 | 126 |
|  | 2,6 | 2.47 | **0.0074** | **0.014** | 126 |  |  | 2017,2019 | 1.03 | 0.4377 | 0.445 | 126 |
|  | 2,13 | 2.17 | **0.0082** | **0.014** | 126 |  |  | 2017,2020 | 1.78 | **0.0072** | **0.014** | 126 |
|  | 3,4 | 2.01 | **0.008** | **0.014** | 126 |  |  | 2018,2019 | 1.15 | 0.1784 | 0.192 | 126 |
|  | 3,6 | 2.62 | **0.0059** | **0.014** | 126 |  |  | 2018,2020 | 2.03 | **0.0079** | **0.014** | 126 |
|  | 3,13 | 2.35 | **0.0103** | **0.015** | 126 |  |  | 2019,2020 | 1.29 | 0.0573 | 0.067 | 126 |
|  | 4,6 | 2.31 | **0.0078** | **0.014** | 126 |  | 3 | 2011,2012 | 1.60 | **0.0073** | **0.014** | 126 |
|  | 4,13 | 1.58 | **0.015** | **0.021** | 126 |  |  | 2011,2013 | 1.69 | **0.0146** | **0.02** | 126 |
|  | 6,13 | 1.82 | **0.0061** | **0.014** | 126 |  |  | 2011,2017 | 1.91 | **0.0069** | **0.014** | 126 |
| 2017 | 1,2 | 2.42 | **0.0078** | **0.014** | 126 |  |  | 2011,2018 | 2.09 | **0.0077** | **0.014** | 126 |
|  | 1,3 | 2.37 | **0.0103** | **0.015** | 126 |  |  | 2011,2019 | 1.69 | **0.0079** | **0.014** | 126 |
|  | 1,4 | 2.93 | **0.0074** | **0.014** | 126 |  |  | 2011,2020 | 2.20 | **0.0086** | **0.014** | 126 |
|  | 1,6 | 2.48 | **0.009** | **0.014** | 126 |  |  | 2012,2013 | 1.28 | 0.0751 | 0.086 | 126 |
|  | 1,13 | 2.50 | **0.0088** | **0.014** | 126 |  |  | 2012,2017 | 1.40 | **0.0339** | **0.042** | 126 |
|  | 2,3 | 1.18 | 0.1974 | 0.21 | 126 |  |  | 2012,2018 | 1.61 | **0.0178** | **0.023** | 126 |
|  | 2,4 | 1.61 | **0.0086** | **0.014** | 126 |  |  | 2012,2019 | 1.53 | **0.0164** | **0.022** | 126 |
|  | 2,6 | 2.19 | **0.0067** | **0.014** | 126 |  |  | 2012,2020 | 1.67 | **0.0079** | **0.014** | 126 |
|  | 2,13 | 2.04 | **0.0072** | **0.014** | 126 |  |  | 2013,2017 | 1.40 | **0.0353** | **0.043** | 126 |
|  | 3,4 | 2.17 | **0.0072** | **0.014** | 126 |  |  | 2013,2018 | 1.57 | **0.0102** | **0.015** | 126 |
|  | 3,6 | 2.19 | **0.0085** | **0.014** | 126 |  |  | 2013,2019 | 1.47 | **0.0413** | 0.05 | 126 |
|  | 3,13 | 2.04 | **0.0079** | **0.014** | 126 |  |  | 2013,2020 | 1.85 | **0.006** | **0.014** | 126 |
|  | 4,6 | 2.25 | **0.0078** | **0.014** | 126 |  |  | 2017,2018 | 1.14 | 0.2554 | 0.269 | 125 |
|  | 4,13 | 1.68 | **0.0065** | **0.014** | 126 |  |  | 2017,2019 | 1.50 | **0.0179** | **0.023** | 126 |
|  | 6,13 | 1.57 | **0.0161** | **0.021** | 126 |  |  | 2017,2020 | 1.36 | 0.0628 | 0.073 | 126 |
|  |  |  |  |  |  |  |  | 2018,2019 | 1.19 | 0.1714 | 0.185 | 126 |
|  |  |  |  |  |  |  |  | 2018,2020 | 1.00 | 0.4085 | 0.419 | 126 |
|  |  |  |  |  |  |  |  | 2019,2020 | 1.32 | 0.0905 | 0.102 | 126 |

Pairwise PERMANOVA comparisons, continued

| Year | Sites | *t* | *p* (perm) | *p* (adj) | Perms |  | Site | Years | *t* | *p* (perm) | *p* (adj) | Perms |
| --- | --- | --- | --- | --- | --- | --- | --- | --- | --- | --- | --- | --- |
| 2018 | 1,2 | 2.48 | **0.0081** | **0.014** | 126 |  | 4 | 2011,2012 | 1.40 | **0.0022** | **0.014** | 462 |
|  | 1,3 | 2.51 | **0.0073** | **0.014** | 126 |  |  | 2011,2013 | 1.02 | 0.4208 | 0.43 | 462 |
|  | 1,4 | 2.63 | **0.0096** | **0.014** | 126 |  |  | 2011,2017 | 2.11 | **0.0023** | **0.014** | 462 |
|  | 1,6 | 2.33 | **0.0072** | **0.014** | 126 |  |  | 2011,2018 | 2.08 | **0.0026** | **0.014** | 462 |
|  | 1,13 | 2.64 | **0.0083** | **0.014** | 126 |  |  | 2011,2019 | 2.48 | **0.0023** | **0.014** | 462 |
|  | 2,3 | 1.19 | 0.1121 | 0.124 | 126 |  |  | 2011,2020 | 2.52 | **0.002** | **0.014** | 462 |
|  | 2,4 | 1.77 | **0.0096** | **0.014** | 126 |  |  | 2012,2013 | 1.26 | **0.016** | **0.021** | 126 |
|  | 2,6 | 2.06 | **0.0075** | **0.014** | 126 |  |  | 2012,2017 | 2.26 | **0.0078** | **0.014** | 126 |
|  | 2,13 | 2.81 | **0.0082** | **0.014** | 126 |  |  | 2012,2018 | 2.12 | **0.0083** | **0.014** | 126 |
|  | 3,4 | 2.19 | **0.0091** | **0.014** | 126 |  |  | 2012,2019 | 2.49 | **0.0076** | **0.014** | 126 |
|  | 3,6 | 2.51 | **0.0073** | **0.014** | 126 |  |  | 2012,2020 | 2.49 | **0.0089** | **0.014** | 126 |
|  | 3,13 | 3.35 | **0.0065** | **0.014** | 126 |  |  | 2013,2017 | 2.11 | **0.0077** | **0.014** | 126 |
|  | 4,6 | 1.69 | **0.0088** | **0.014** | 126 |  |  | 2013,2018 | 1.90 | **0.0063** | **0.014** | 126 |
|  | 4,13 | 1.73 | **0.0081** | **0.014** | 126 |  |  | 2013,2019 | 2.24 | **0.0079** | **0.014** | 126 |
|  | 6,13 | 1.85 | **0.0059** | **0.014** | 126 |  |  | 2013,2020 | 2.38 | **0.0081** | **0.014** | 126 |
| 2019 | 1,2 | 1.64 | **0.0149** | **0.02** | 126 |  |  | 2017,2018 | 1.08 | 0.3176 | 0.331 | 126 |
|  | 1,3 | 1.85 | **0.0073** | **0.014** | 126 |  |  | 2017,2019 | 1.72 | **0.0079** | **0.014** | 126 |
|  | 1,4 | 2.17 | **0.0078** | **0.014** | 126 |  |  | 2017,2020 | 1.77 | **0.0069** | **0.014** | 126 |
|  | 1,6 | 2.07 | **0.0064** | **0.014** | 126 |  |  | 2018,2019 | 1.16 | 0.1159 | 0.128 | 126 |
|  | 1,13 | 2.19 | **0.008** | **0.014** | 126 |  |  | 2018,2020 | 1.46 | **0.0106** | **0.015** | 126 |
|  | 2,3 | 1.29 | 0.0872 | 0.099 | 126 |  |  | 2019,2020 | 1.45 | **0.0089** | **0.014** | 126 |
|  | 2,4 | 1.71 | **0.0069** | **0.014** | 126 |  | 13 | 2011,2012 | 1.08 | 0.3063 | 0.322 | 126 |
|  | 2,6 | 1.95 | **0.0078** | **0.014** | 126 |  |  | 2011,2013 | 1.23 | 0.0865 | 0.099 | 126 |
|  | 2,13 | 1.93 | **0.0083** | **0.014** | 126 |  |  | 2011,2017 | 1.42 | **0.0486** | 0.058 | 126 |
|  | 3,4 | 2.38 | **0.0093** | **0.014** | 126 |  |  | 2011,2018 | 1.70 | **0.0076** | **0.014** | 126 |
|  | 3,6 | 2.73 | **0.0093** | **0.014** | 126 |  |  | 2011,2019 | 1.76 | **0.0079** | **0.014** | 126 |
|  | 3,13 | 3.03 | **0.0074** | **0.014** | 126 |  |  | 2011,2020 | 1.92 | **0.0088** | **0.014** | 126 |
|  | 4,6 | 2.40 | **0.0094** | **0.014** | 126 |  |  | 2012,2013 | 1.55 | **0.0078** | **0.014** | 126 |
|  | 4,13 | 2.21 | **0.0082** | **0.014** | 126 |  |  | 2012,2017 | 1.48 | **0.0385** | **0.047** | 126 |
|  | 6,13 | 2.02 | **0.0083** | **0.014** | 126 |  |  | 2012,2018 | 2.18 | **0.0103** | **0.015** | 126 |
| 2020 | 1,2 | 2.67 | **0.0091** | **0.014** | 126 |  |  | 2012,2019 | 1.92 | **0.0072** | **0.014** | 126 |
|  | 1,3 | 2.75 | **0.0084** | **0.014** | 126 |  |  | 2012,2020 | 2.08 | **0.0105** | **0.015** | 126 |
|  | 1,4 | 3.61 | **0.0101** | **0.015** | 126 |  |  | 2013,2017 | 1.25 | 0.0744 | 0.086 | 126 |
|  | 1,6 | 3.22 | **0.0078** | **0.014** | 126 |  |  | 2013,2018 | 1.49 | **0.0148** | 0.02 | 126 |
|  | 1,13 | 3.43 | **0.0071** | **0.014** | 126 |  |  | 2013,2019 | 1.39 | **0.0176** | **0.023** | 126 |
|  | 2,3 | 1.21 | 0.1536 | 0.167 | 126 |  |  | 2013,2020 | 1.54 | **0.0161** | **0.021** | 126 |
|  | 2,4 | 3.27 | **0.008** | **0.014** | 126 |  |  | 2017,2018 | 1.37 | **0.0095** | **0.014** | 126 |
|  | 2,6 | 3.12 | **0.009** | **0.014** | 126 |  |  | 2017,2019 | 1.38 | **0.0151** | **0.021** | 126 |
|  | 2,13 | 3.53 | **0.0082** | **0.014** | 126 |  |  | 2017,2020 | 1.36 | **0.0248** | **0.031** | 126 |
|  | 3,4 | 2.39 | **0.009** | **0.014** | 126 |  |  | 2018,2019 | 1.34 | **0.0272** | **0.034** | 126 |
|  | 3,6 | 2.72 | **0.0077** | **0.014** | 126 |  |  | 2018,2020 | 1.53 | **0.0085** | **0.014** | 126 |
|  | 3,13 | 2.71 | **0.0076** | **0.014** | 126 |  |  | 2019,2020 | 1.29 | 0.0981 | 0.111 | 126 |
|  | 4,6 | 2.38 | **0.0074** | **0.014** | 126 |  | 6 | 2011,2012 | 1.03 | 0.3974 | 0.41 | 1703 |
|  | 4,13 | 1.57 | **0.0088** | **0.014** | 126 |  |  | 2011,2013 | 1.31 | **0.0434** | 0.052 | 462 |
|  | 6,13 | 2.02 | **0.0084** | **0.014** | 126 |  |  | 2011,2017 | 1.10 | 0.2451 | 0.26 | 462 |
|  |  |  |  |  |  |  |  | 2011,2018 | 1.48 | **0.006** | **0.014** | 462 |
|  |  |  |  |  |  |  |  | 2011,2019 | 2.36 | **0.0019** | **0.014** | 462 |
|  |  |  |  |  |  |  |  | 2011,2020 | 1.16 | 0.1905 | 0.204 | 462 |
|  |  |  |  |  |  |  |  | 2012,2013 | 1.93 | **0.0015** | **0.014** | 792 |
|  |  |  |  |  |  |  |  | 2012,2017 | 1.58 | **0.0021** | **0.014** | 791 |
|  |  |  |  |  |  |  |  | 2012,2018 | 1.97 | **0.0011** | **0.014** | 792 |
|  |  |  |  |  |  |  |  | 2012,2019 | 2.95 | **0.0014** | **0.014** | 792 |
|  |  |  |  |  |  |  |  | 2012,2020 | 1.35 | 0.0592 | 0.069 | 792 |
|  |  |  |  |  |  |  |  | 2013,2017 | 1.37 | **0.0383** | **0.047** | 126 |
|  |  |  |  |  |  |  |  | 2013,2018 | 1.19 | 0.1306 | 0.143 | 126 |
|  |  |  |  |  |  |  |  | 2013,2019 | 1.88 | **0.0084** | **0.014** | 126 |
|  |  |  |  |  |  |  |  | 2013,2020 | 1.83 | **0.0081** | **0.014** | 126 |
|  |  |  |  |  |  |  |  | 2017,2018 | 1.29 | 0.1053 | 0.118 | 126 |
|  |  |  |  |  |  |  |  | 2017,2019 | 2.22 | **0.0075** | **0.014** | 126 |
|  |  |  |  |  |  |  |  | 2017,2020 | 1.37 | **0.0239** | **0.03** | 126 |
|  |  |  |  |  |  |  |  | 2018,2019 | 1.68 | **0.0089** | **0.014** | 126 |
|  |  |  |  |  |  |  |  | 2018,2020 | 1.71 | **0.0186** | **0.024** | 126 |
|  |  |  |  |  |  |  |  | 2019,2020 | 2.42 | **0.0091** | **0.014** | 126 |

Supplementary Table S6. Mean (SE) and median (***bold, italicised***) abundances across all fall sampling dates (n = 7) for 17 taxa selected by BVSTEP and 3 taxa (X and Y) identified via SIMPER analyses. TC = Taxon Classification: P = Polychaeta; MB = Mollusca, Bivalvia; MG = Mollusca, Gastropoda; CC = Crustacea, Cumacea; CA = Crustacea, Amphipoda. EG – ecological group assigned in AMBI.

| ID | Species / Taxon | TC | EG | SITE 1 | SITE 2 | SITE 3 | SITE 4 | SITE 13 | SITE 6 |
| --- | --- | --- | --- | --- | --- | --- | --- | --- | --- |
| A | *Cossura longocirrata* | P | IV | 24.07  (9.47) | 308.37  (166.27) | 349.54  (136.67) | 28.04  (6.57) | 4.89  (3.28) | 2.54  (1.51) |
|  |  |  |  | ***15.8*** | ***136.6*** | ***225.8*** | ***31.6*** | ***1.8*** | ***0.8*** |
| B | *Levinsenia gracilis* | P | III | 2.11 (1.44) | 46.81 (5.87) | 32.29 (7.57) | 18.78 (4.8) | 6.34  (1.83) | 15.36  (4.55) |
|  |  |  |  | ***0.2*** | ***50.6*** | ***21.4*** | ***12*** | ***6.2*** | ***12.7*** |
| C | *Tharyx* spp. and *Chaetozone* spp. | P | III | 5.91 (1.87) | 33.79 (5.76) | 78.14 (14.5) | 82.84 (17.72) | 31.31  (5.87) | 13.54  (8.14) |
|  |  |  |  | ***6.6*** | ***31.6*** | ***70.6*** | ***102.6*** | ***31.4*** | ***7.2*** |
| D | *Nephtys incisa* | P | II | 1.13 (0.31) | 9.31 (2.28) | 5.29 (0.86) | 8.58 (1.21) | 7.6  (0.93) | 4.16  (0.6) |
|  |  |  |  | ***1*** | ***9.2*** | ***5.8*** | ***6.8*** | ***7.4*** | ***4.2*** |
| E | *Aricidea catherinae* | P | I | 1.6 (0.55) | 7.26 (1.22) | 27.66 (5.02) | 10.52 (1.73) | 0.94  (0.27) | 1.02  (0.62) |
|  |  |  |  | ***1.4*** | ***7.8*** | ***29.8*** | ***10.8*** | ***0.8*** | ***0.2*** |
| F | *Ninoe nigripes* | P | III | 0.28 (0.19) | 5.46  (0.8) | 3.51 (0.47) | 7.05 (1.81) | 3.37  (0.54) | 4.61  (0.92) |
|  |  |  |  | ***0.1*** | ***6*** | ***4*** | ***6.6*** | ***3*** | ***3.8*** |
| G | *Sternaspis scutata* | P | III | 1.01 (0.56) | 4.23 (1.16) | 4.29 (1.25) | 0.9  (0.21) | 0.37  (0.15) | 0.2  (0.09) |
|  |  |  |  | ***0.4*** | ***4*** | ***3.4*** | ***1*** | ***0.2*** | ***0.2*** |
| H | *Terebellides stroemii* | P | II | 0  (0) | 1.14 (0.59) | 0.49  (0.2) | 6  (3.12) | 6.34  (3.19) | 2.76  (1.46) |
|  |  |  |  | ***0*** | ***0.8*** | ***0.6*** | ***2*** | ***3*** | ***0.8*** |
| I | *Sabellaria vulgaris* | P | I | 0  (0) | 0  (0) | 0  (0) | 0.03 (0.03) | 0  (0) | 2.34  (0.69) |
|  |  |  |  | ***0*** | ***0*** | ***0*** | ***0*** | ***0*** | ***2.3*** |
| J | *Nucula proxima* | MB | I | 0.23 (0.13) | 34.34 (15.79) | 13.14 (3.98) | 45.34 (6.34) | 91.83  (10.03) | 79.84  (49.79) |
|  |  |  |  | ***0.2*** | ***20.4*** | ***11.2*** | ***48.8*** | ***92*** | ***20*** |
| K | *Ennucula delphinodonta* | MB | II | 0.02 (0.02) | 2.04 (0.58) | 3.34 (1.18) | 10.94 (2.89) | 4.11  (1.12) | 0.93  (0.34) |
|  |  |  |  | ***0*** | ***2.4*** | ***2.4*** | ***11*** | ***3.8*** | ***0.6*** |
| L | *Kurtiella planulata* | MB | III | 0.08 (0.06) | 1.17 (0.59) | 0.97 (0.43) | 2.36 (0.72) | 1.2  (0.36) | 1.32  (0.64) |
|  |  |  |  | ***0*** | ***0.6*** | ***0.4*** | ***1.8*** | ***1.4*** | ***0.6*** |
| M | *Ilyanassa trivittata* | MG | II | 0.27 (0.14) | 1.81 (0.46) | 1.54 (0.38) | 1.2  (0.39) | 1.8  (0.64) | 3.28  (0.45) |
|  |  |  |  | ***0.2*** | ***1.5*** | ***1.8*** | ***1.8*** | ***1.2*** | ***3.4*** |
| N | *Eudorella truncatula* | CC | I | 0  (0) | 0.2  (0.14) | 0.17 (0.14) | 0.69 (0.31) | 0.57  (0.2) | 1.96  (0.83) |
|  |  |  |  | ***0*** | ***0*** | ***0*** | ***0.2*** | ***0.6*** | ***0.8*** |
| O | *Diastylis* spp. (mostly *D. sculpta*) | CC | II | 0.72 (0.56)  ***0*** | 2.39 (0.55)  ***2*** | 3.54 (1.46)  ***2.4*** | 0.91 (0.33)  ***0.6*** | 0.49  (0.17)  ***0.2*** | 0.45  (0.21)  ***0.2*** |
|  |  |  |  |  |  |  |  |  |  |
| P | U*nciola irrorata* | CA | I | 0  (0) | 0  (0) | 0.09 (0.06) | 0.09 (0.09) | 0.06  (0.04) | 2.73  (1.17) |
|  |  |  |  | ***0*** | ***0*** | ***0*** | ***0*** | ***0*** | ***1.8*** |
| Q | Nematoda | N | III | 3.22 (1.32) | 1.15 (0.42) | 3.06  (1) | 1.16 (0.47) | 1.31  (0.42) | 2.61  (1.91) |
|  |  |  |  | ***1.6*** | ***0.4*** | ***2.6*** | ***0.4*** | ***1*** | ***0.8*** |
| X | *Clymenella torquata* and other Maldanidae | P | I | 5.19  (3.76)  ***1.1*** | 0.31  (0.11)  ***0.2*** | 0.89  (0.26)  ***0.8*** | 0.09  (0.04)  ***0*** | 0.03  (0.03)  ***0*** | 0.65  (0.27)  ***0.3*** |
|  |  |  |  |  |  |  |  |  |  |
| Y | *Eteone longa* | P | III | 0.17 (0.07) | 3.26 (0.77) | 2.31 (0.61) | 1.22 (0.46) | 0.29  (0.16) | 0.17  (0.09) |
|  |  |  |  | ***0.2*** | ***2.6*** | ***2*** | ***1.4*** | ***0.2*** | ***0*** |
| Z | *Thyasira flexuosa* | MB | III | 0  (0) | 0.37 (0.23) | 0.37 (0.15) | 1.23 (0.41) | 1.57  (0.3) | 1.62  (0.49) |
|  |  |  |  | ***0*** | ***0*** | ***0.2*** | ***1.4*** | ***1.6*** | ***1.2*** |

Supplementary Table S7. Principal Components Analysis, UNB metals data, all seasons 2011-2018

1. Variance explained by first 10 principal components

|  | PC1 | PC2 | PC3 | PC4 | PC5 | PC6 | PC7 | PC8 | PC9 | PC10 |
| --- | --- | --- | --- | --- | --- | --- | --- | --- | --- | --- |
| Standard deviation | 3.270 | 1.233 | 0.976 | 0.914 | 0.796 | 0.710 | 0.673 | 0.565 | 0.498 | 0.477 |
| Proportion of Variance | 0.629 | 0.089 | 0.056 | 0.049 | 0.037 | 0.030 | 0.027 | 0.019 | 0.015 | 0.013 |
| Cumulative Proportion | 0.629 | 0.718 | 0.775 | 0.824 | 0.861 | 0.891 | 0.917 | 0.936 | 0.951 | 0.964 |

1. Metal loadings for first 3 principal components

|  | PC1 | PC2 | PC3 |
| --- | --- | --- | --- |
| Al | 0.269 | -0.012 | 0.050 |
| As | 0.270 | 0.014 | 0.025 |
| Co | 0.282 | 0.138 | 0.010 |
| Cr | 0.289 | -0.089 | -0.124 |
| Cu | 0.235 | 0.039 | 0.089 |
| Fe | 0.240 | 0.144 | 0.226 |
| La | 0.100 | -0.597 | 0.356 |
| Mg | 0.266 | 0.092 | 0.163 |
| Mn | 0.227 | -0.284 | 0.300 |
| Ni | 0.289 | 0.113 | -0.087 |
| P | 0.151 | -0.455 | -0.185 |
| Pb | 0.183 | 0.334 | 0.516 |
| Rb | 0.218 | -0.125 | -0.528 |
| S | 0.210 | 0.382 | -0.228 |
| Sr | 0.242 | -0.096 | -0.059 |
| V | 0.283 | -0.060 | -0.094 |
| Zn | 0.278 | 0.017 | -0.169 |

Supplementary Table S8. Principal Components analysis of RPC metals data, 2018-2021

1. Variance explained by first 10 principal components

|  | PC1 | PC2 | PC3 | PC4 | PC5 | PC6 | PC7 | PC8 | PC9 | PC10 |
| --- | --- | --- | --- | --- | --- | --- | --- | --- | --- | --- |
| Standard deviation | 4.112 | 1.099 | 0.887 | 0.619 | 0.481 | 0.363 | 0.321 | 0.243 | 0.226 | 0.185 |
| Proportion of Variance | 0.846 | 0.060 | 0.039 | 0.019 | 0.012 | 0.007 | 0.005 | 0.003 | 0.003 | 0.002 |
| Cumulative Proportion | 0.846 | 0.906 | 0.945 | 0.964 | 0.976 | 0.983 | 0.988 | 0.991 | 0.993 | 0.995 |

1. Metal loadings for first 3 principal components

|  | PC1 | PC2 | PC3 |
| --- | --- | --- | --- |
| Al | 0.239 | -0.085 | 0.104 |
| As | 0.203 | 0.222 | 0.028 |
| Ba | -0.045 | -0.725 | -0.638 |
| B | 0.237 | 0.004 | -0.093 |
| Ca | 0.134 | 0.532 | -0.619 |
| Co | 0.235 | -0.081 | 0.112 |
| Cr | 0.240 | -0.074 | 0.060 |
| Cu | 0.236 | -0.037 | -0.006 |
| Fe | 0.238 | -0.083 | 0.123 |
| K | 0.238 | -0.093 | 0.063 |
| Li | 0.240 | -0.054 | 0.095 |
| Mg | 0.238 | 0.003 | 0.025 |
| Mn | 0.217 | 0.003 | -0.033 |
| Na | 0.228 | 0.034 | -0.133 |
| Ni | 0.238 | -0.070 | 0.093 |
| Pb | 0.235 | -0.106 | -0.055 |
| Rb | 0.239 | -0.067 | 0.105 |
| Sr | 0.218 | 0.260 | -0.319 |
| V | 0.239 | -0.105 | -0.015 |
| Zn | 0.239 | -0.051 | 0.043 |

Supplemental Table S9. Summary of Partial Least Squares Regression (PLSR) model Components, UNB data (2011-2013 + 2018). Important variables (VIP > 1) highlighted in bold, variables with overall negative relationships with the response variable highlighted in italic font. Prefix ‘lg’ in variables names indicates that variables were log transformed prior to analysis.

a) Abundance (log transformed); variance explained by PLSR model = 48.76%

|  | Component 1 | Component 2 | Component 3 |
| --- | --- | --- | --- |
| Proportion of variance in X | 37.96 | 15.53 | 4.35 |
| Proportion of variance in Y | 19.63 | 21.95 | 7.18 |
| Variable loadings |  |  |  |
| lgDEPTH | -0.02841 | 0.135957 | 0.257001 |
| lgAl | 0.314994 | -0.2741 | 0.385085 |
| lgAs | 0.368263 | -0.25621 | 0.030969 |
| lgCr | 0.376886 | -0.28003 | 0.201462 |
| Cu | 0.349336 | -0.1217 | -0.50642 |
| lgFe | 0.302232 | -0.31336 | 0.130298 |
| **lgLa** | **0.009228** | **-0.4299** | **0.180523** |
| lgMg | 0.342119 | -0.26623 | -0.03718 |
| **lgMn** | **0.247186** | **-0.38648** | **-0.02976** |
| P | 0.198115 | -0.26313 | -0.00315 |
| lgPb | 0.360566 | -0.06167 | -0.07095 |
| Rb | 0.271194 | -0.02436 | 0.415303 |
| lgS | 0.330791 | -0.1484 | -0.15254 |
| lgSr | 0.331235 | -0.29143 | 0.242403 |
| lgZn | 0.403608 | -0.20773 | -0.06201 |
| lgHg | 0.305503 | -0.03142 | -0.59785 |
| **lgTOC** | **0.376365** | **0.122139** | **-0.05742** |
| lgLOI950 | 0.184535 | -0.10135 | 0.321621 |
| **lgSiltClay** | **0.198563** | **-0.01964** | **0.182477** |
| **lgCoarseSand** | **-0.14136** | **-0.17094** | **0.095677** |
| **lgGranulesPebbles** | **-0.14451** | **-0.19483** | **0.099769** |

b) Species Richness; variance explained by PLSR model = 44.95 %

|  | Component 1 | Component 2 |
| --- | --- | --- |
| Proportion of variance in X | 13.63 | 20.26 |
| Proportion of variance in Y | 37.86 | 7.09 |
| Variable loadings |  |  |
| **lgDEPTH** | **0.542618** | **0.168544** |
| lgAl | 0.121944 | -0.31285 |
| lgAs | 0.06176 | -0.233 |
| lgCr | 0.099853 | -0.39585 |
| Cu | -0.06381 | -0.08124 |
| lgFe | 0.047065 | -0.41882 |
| **lgLa** | **-0.40337** | **-0.22418** |
| lgMg | 0.089638 | -0.36743 |
| lgMn | -0.13191 | -0.23616 |
| **P** | **-0.32745** | **-0.28487** |
| lgPb | 0.098335 | 0.038847 |
| Rb | 0.284627 | -0.35473 |
| lgS | 0.303429 | -0.33445 |
| lgSr | 0.066711 | -0.27464 |
| lgZn | 0.174739 | -0.31338 |
| lgHg | -0.03912 | -0.21937 |
| lgTOC | 0.152607 | -0.22129 |
| lgLOI950 | 0.177184 | -0.41037 |
| **lgSiltClay** | **-0.36288** | **0.176843** |
| lgCoarseSand | 0.176676 | -0.42044 |
| **lgGranulesPebbles** | **0.350301** | **-0.25128** |

c) Shannon Diversity; variance explained by PLSR model = 57.09 %

|  | Component 1 | Component 2 | Component 3 |
| --- | --- | --- | --- |
| Proportion of variance in X | 40.8 | 17.34 | 11.62 |
| Proportion of variance in Y | 30.31 | 21.65 | 5.13 |
| Variable loadings |  |  |  |
| **lgDEPTH** | **0.199689** | **0.413866** | **-0.01131** |
| lgAl | -0.26536 | 0.315182 | -0.00439 |
| lgAs | -0.31555 | 0.256812 | 0.08022 |
| lgCr | -0.32728 | 0.265573 | -0.11021 |
| Cu | -0.28978 | 0.081795 | 0.179855 |
| lgFe | -0.28727 | 0.242385 | -0.08258 |
| lgLa | -0.17394 | -0.04862 | 0.234355 |
| lgMg | -0.29764 | 0.256049 | -0.06061 |
| lgMn | -0.29586 | 0.166658 | 0.177359 |
| **P** | **-0.30854** | **-0.13769** | **-0.02395** |
| lgPb | -0.24162 | 0.229648 | 0.258171 |
| Rb | -0.18224 | 0.213144 | -0.41099 |
| lgS | -0.21959 | 0.336293 | -0.20339 |
| lgSr | -0.29207 | 0.271801 | 0.046751 |
| lgZn | -0.31366 | 0.295419 | -0.07189 |
| **lgHg** | **-0.28163** | **-0.0445** | **-0.16777** |
| lgTOC | -0.26128 | 0.054907 | -0.25878 |
| lgLOI950 | -0.16058 | 0.136547 | -0.39557 |
| lgSiltClay | -0.20032 | -0.18776 | 0.417925 |
| lgCoarseSand | 0.071814 | 0.114399 | -0.50692 |
| **lgGranulesPebbles** | **0.166693** | **0.355034** | **-0.22673** |

d) AMBI; variance explained by PLSR model = 65.38 %

|  | Comp. 1 | Comp. 2 | Comp. 3 | Comp. 4 | Comp. 5 |
| --- | --- | --- | --- | --- | --- |
| Proportion of variance in X | 42.36 | 15.72 | 9.92 | 3.82 | 2.15 |
| Proportion of variance in Y | 31.00 | 18.31 | 7.55 | 6.41 | 2.11 |
| Variable loadings |  |  |  |  |  |
| **lgDEPTH** | **-0.15046** | **-0.48844** | **-0.0473** | **0.282386** | **-0.13917** |
| lgAl | 0.271559 | -0.25727 | 0.035905 | -0.03484 | 0.33288 |
| lgAs | 0.311859 | -0.18677 | -0.01435 | 0.315716 | -0.29487 |
| lgCr | 0.32052 | -0.23722 | 0.094102 | -0.14699 | 0.008686 |
| Cu | 0.258815 | -0.07163 | -0.32327 | 0.034755 | -0.46966 |
| lgFe | 0.281549 | -0.20045 | 0.0961 | -0.25923 | 0.634313 |
| lgLa | 0.167591 | 0.185632 | -0.14775 | -0.41507 | 0.241492 |
| lgMg | 0.28658 | -0.24454 | 0.014741 | -0.21826 | 0.2682 |
| **lgMn** | **0.294893** | **-0.05057** | **-0.05853** | **0.321587** | **-0.04348** |
| **P** | **0.277257** | **0.239321** | **0.128354** | **-0.02944** | **-0.24942** |
| lgPb | 0.229052 | -0.21949 | -0.38573 | 0.261479 | -0.22697 |
| Rb | 0.185688 | -0.26672 | 0.343356 | -0.20851 | -0.04143 |
| lgS | 0.213245 | -0.38222 | 0.134537 | -0.05737 | -0.12912 |
| **lgSr** | **0.29431** | **-0.18798** | **0.07191** | **0.247805** | **0.209114** |
| lgZn | 0.305225 | -0.29155 | 0.033417 | 0.03137 | -0.22254 |
| lgHg | 0.241685 | 0.00927 | 0.07239 | 0.190979 | 0.004655 |
| lgTOC | 0.235033 | -0.12394 | 0.112089 | -0.21232 | 0.038211 |
| lgLOI950 | 0.156696 | -0.13154 | 0.504306 | -0.29652 | -0.10771 |
| **lgSiltClay** | **0.181608** | **0.296129** | **-0.33927** | **0.335614** | **-0.16155** |
| lgCoarseSand | -0.049 | -0.12862 | 0.613402 | -0.30228 | -0.4311 |
| lgGranulesPebbles | -0.12089 | -0.3332 | 0.416744 | 0.057617 | 0.049426 |

Figure S1. nMDS ordination of reference site samples (Fall data), using Bray-Curtis similarities on fourth-root transformed data. Ordination excludes one sample from Site 1 in 2019 (1.5-2019-11-03) which contained only two individuals and was very separated from the rest of the points.


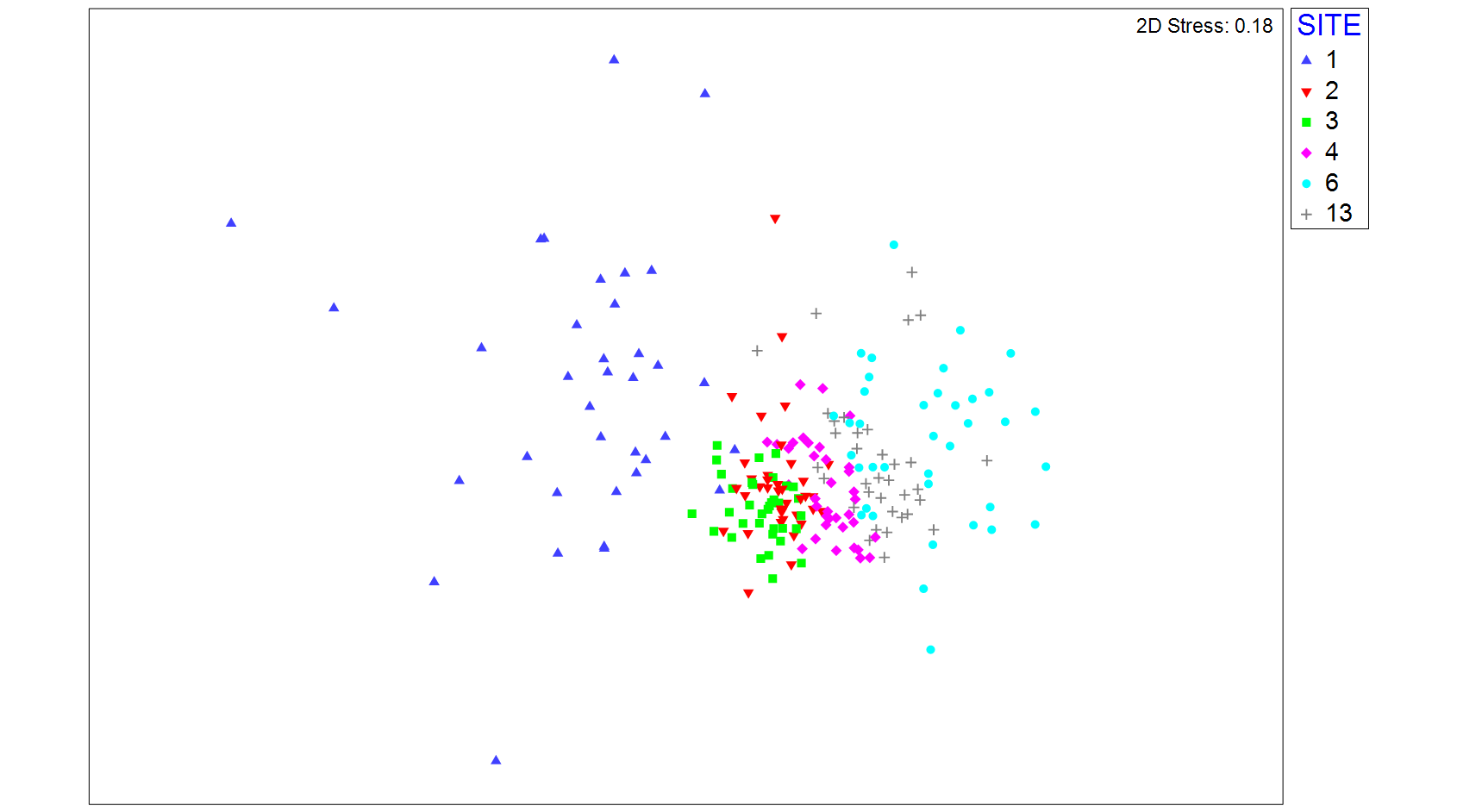


Figure S2. Temporal trends in abundances at Site 1 for 17 species identified by BVSTEP, and 3 species selected following SIMPER analyses.


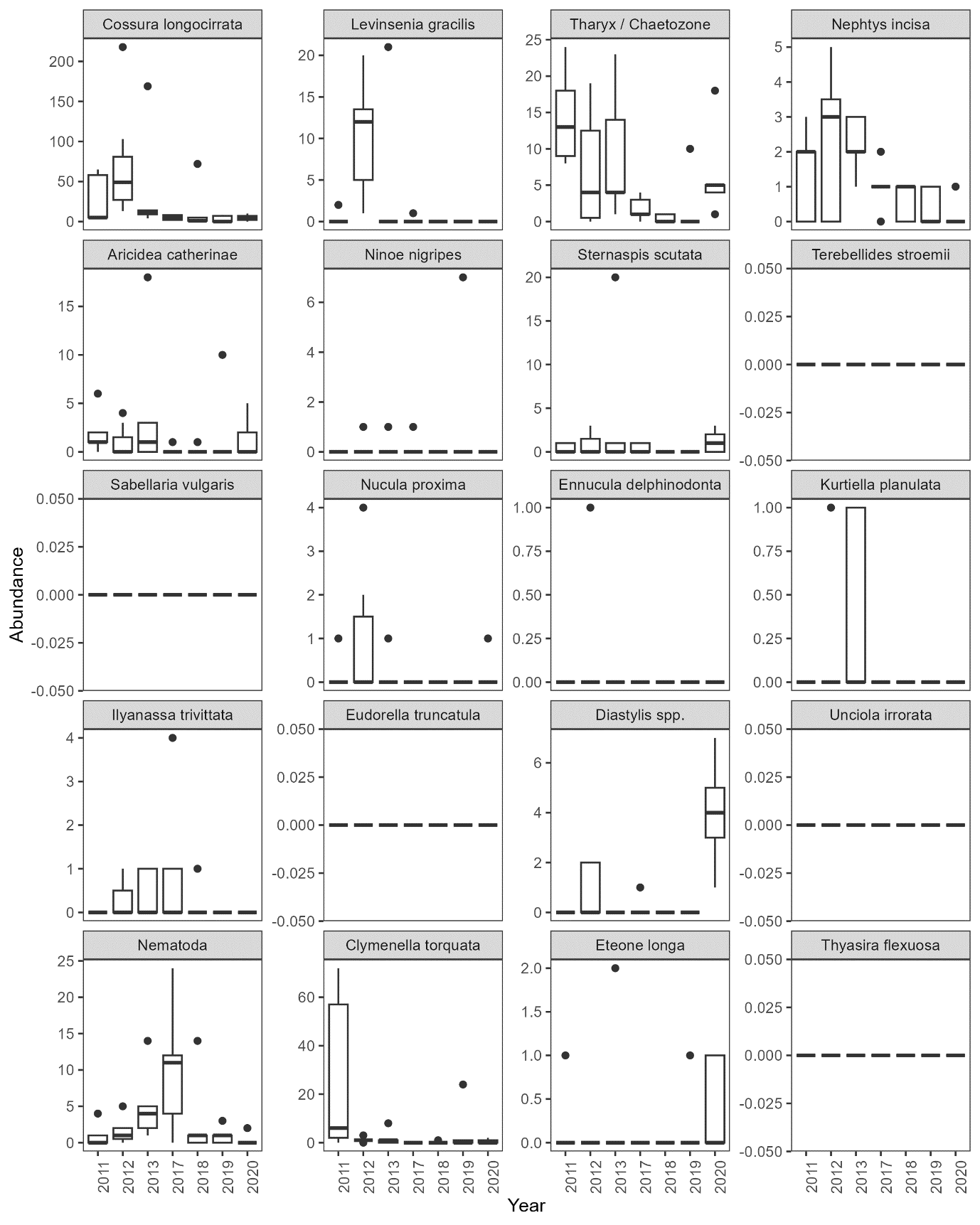


Figure S3. Temporal trends in abundance at Site 2 for 17 species identified by BVSTEP, and 3 species selected following SIMPER analyses.


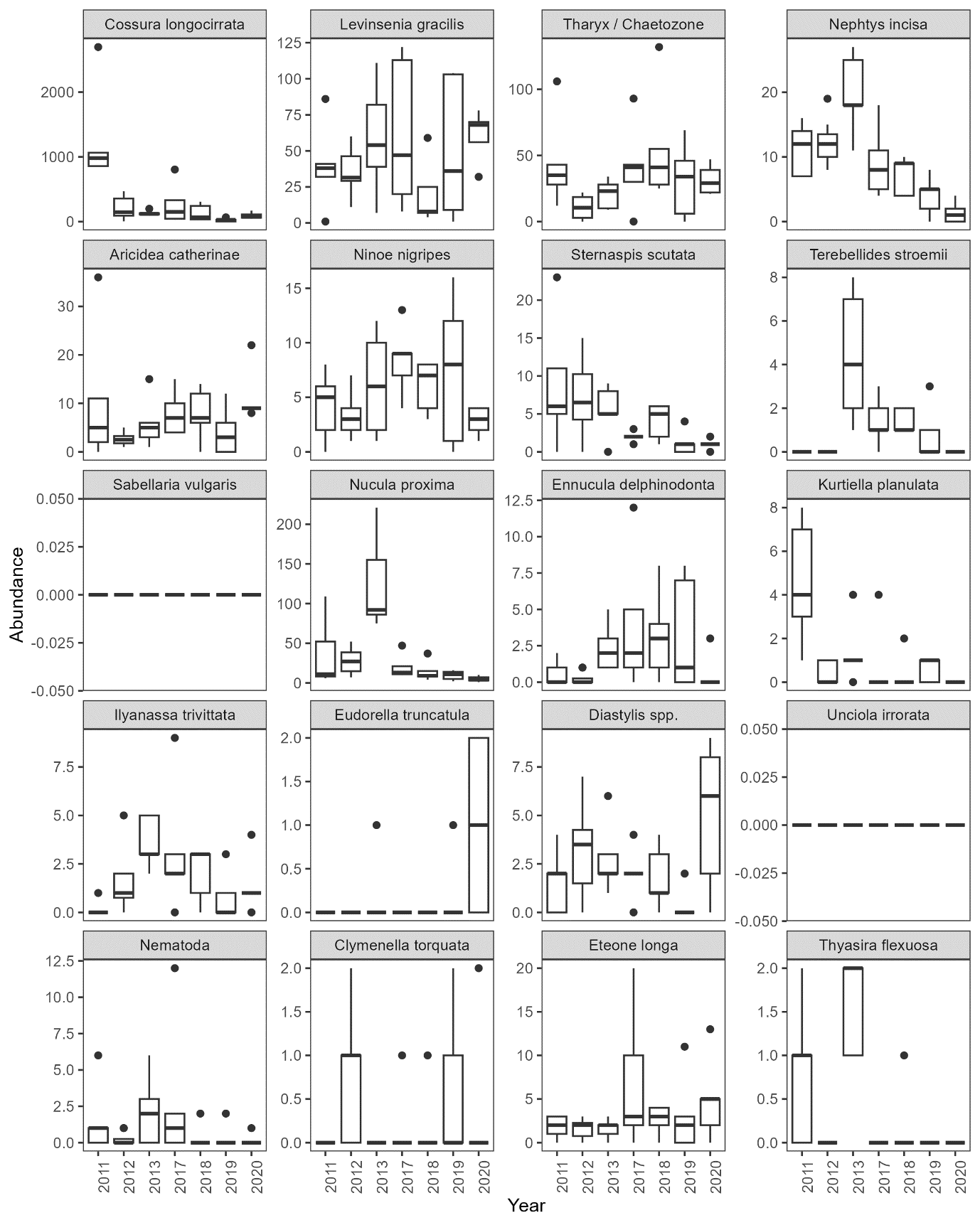


Figure S4. Temporal trends in abundance at Site 3 for 17 species identified by BVSTEP, and 3 species selected following SIMPER analyses.


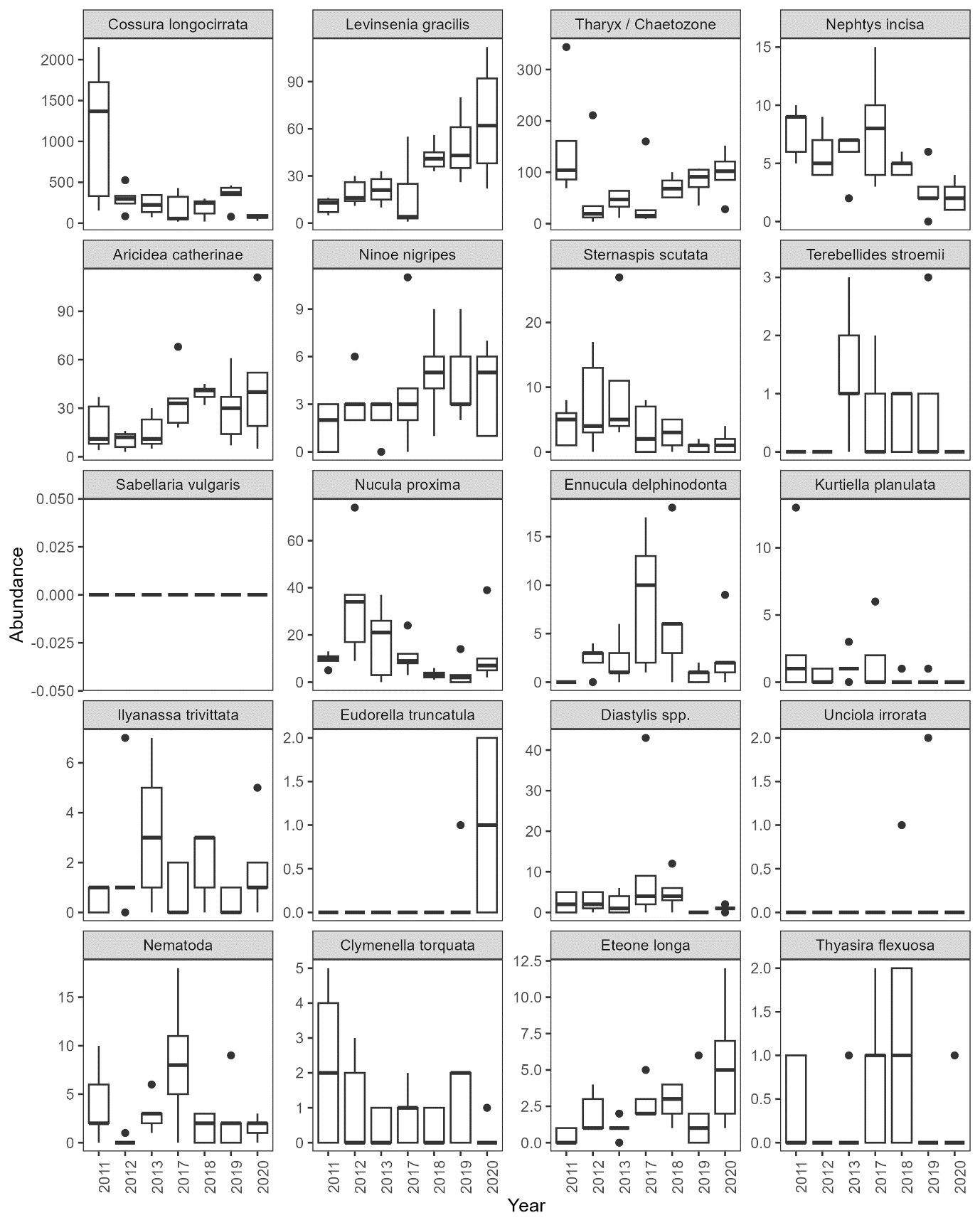


Figure S5. Temporal trends in abundance at Site 4 for 17 species identified by BVSTEP, and 3 species selected following SIMPER analyses.


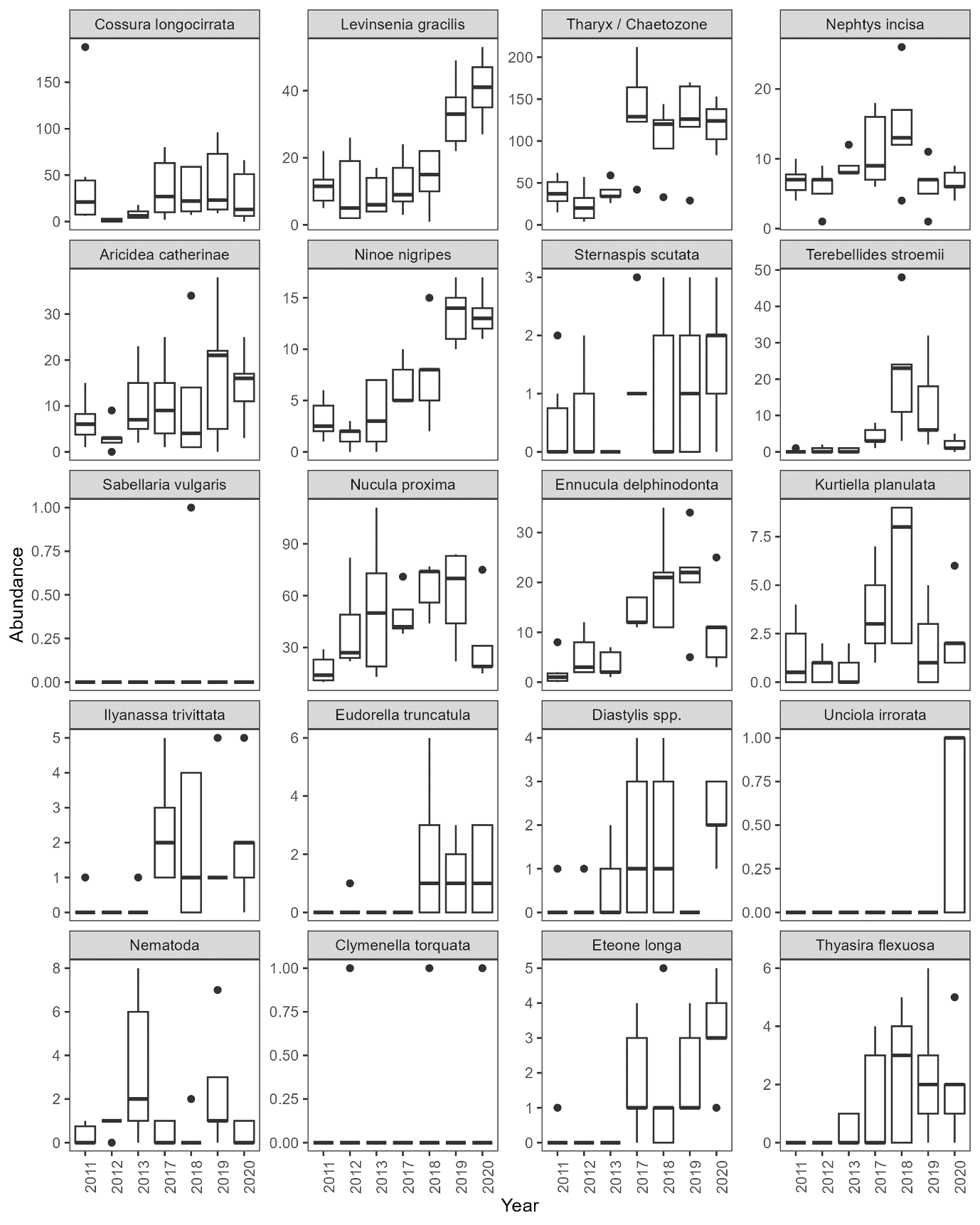


Figure S6. Temporal trends in abundance at Site 13 for 17 species identified by BVSTEP, and 3 species selected following SIMPER analyses.


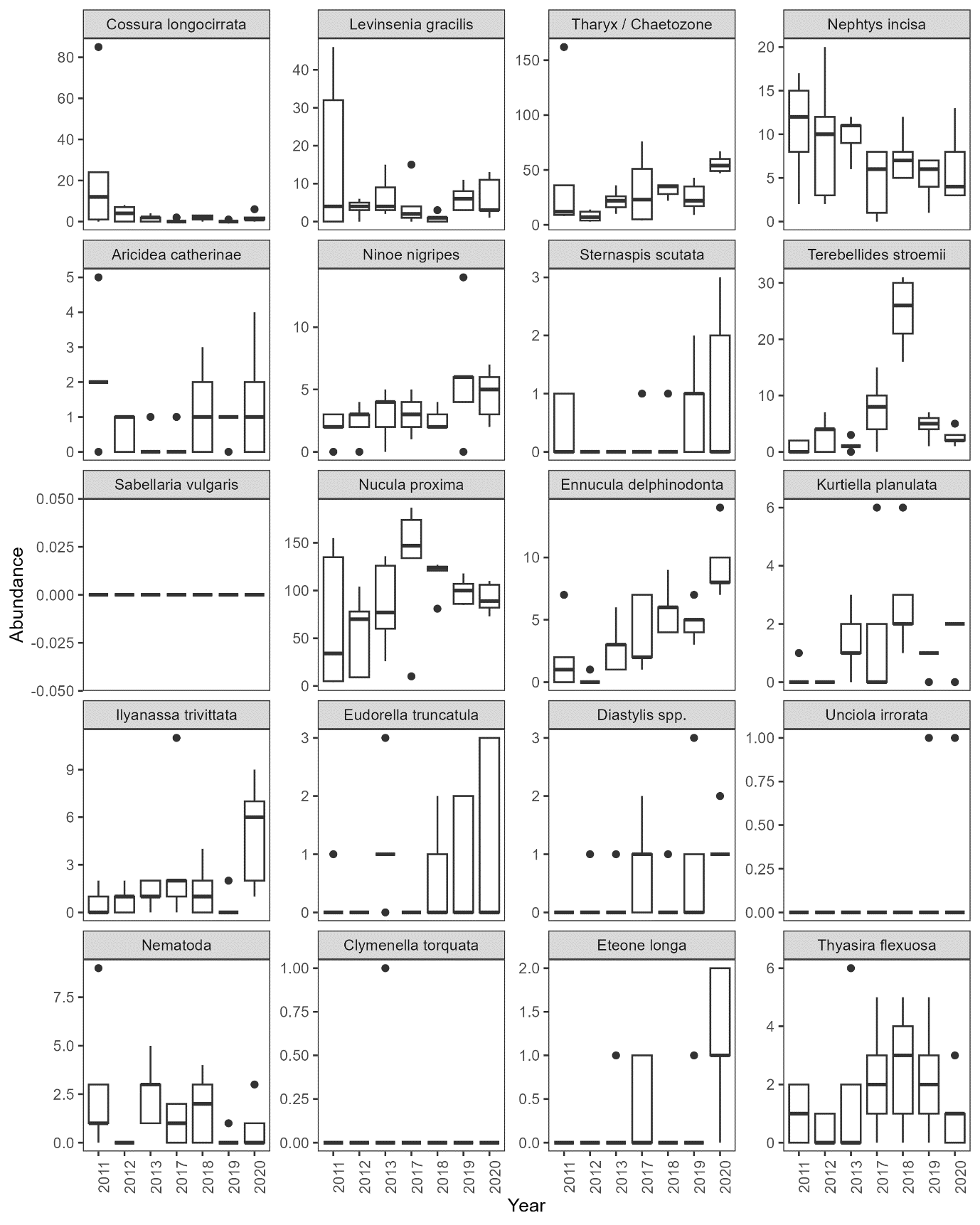


Figure S7. Temporal trends in abundance at Site 6 for 17 species identified by BVSTEP, and 3 species selected following SIMPER analyses.


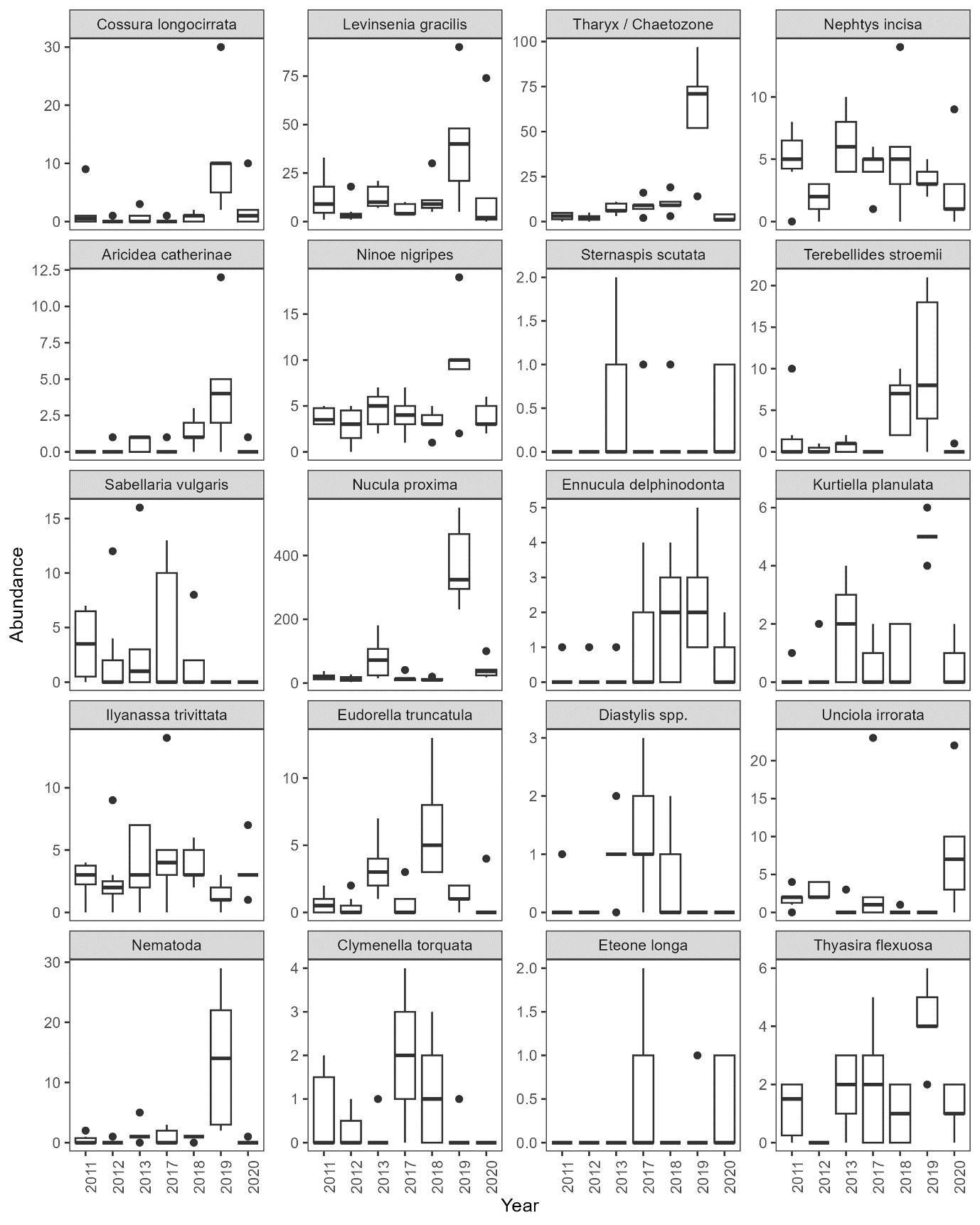


Figure S8. Mean Total Organic Carbon (on all sampling dates) at reference sites. Error bars: ±SE. Dotted lines – ‘normal’ range for each site; Dashed lines – ‘normal’ ranges for Inner Harbour and Outer Harbour; Solid line – normal range for all reference sites. ‘Normal’ ranges are 2SD above and below the grand mean for each site / group of sites and references lines as for Figure 2. 2017 data, and 2020 Site 1 and 2 data, not used for ‘normal’ range calculations.


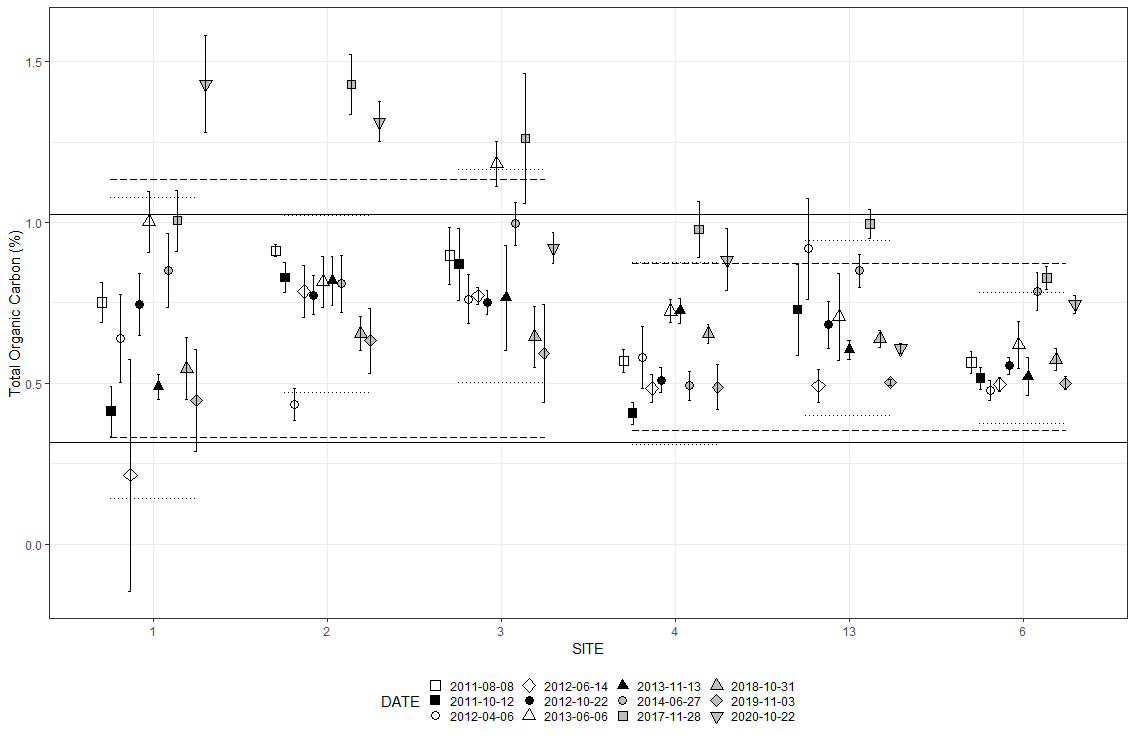


Figure S9. Mean Total PAH concentration (on sampling dates from 2011-2017) at reference sites. Error bars are ±SE. Dotted lines – ‘normal’ range for each site; Solid line – normal range for all reference sites together. ‘Normal’ ranges are 2SD above and below the grand mean for each site / group of sites. Site 1 data for 2013-11-13 were not included in calculations of normal ranges, as they were clearly atypically elevated.


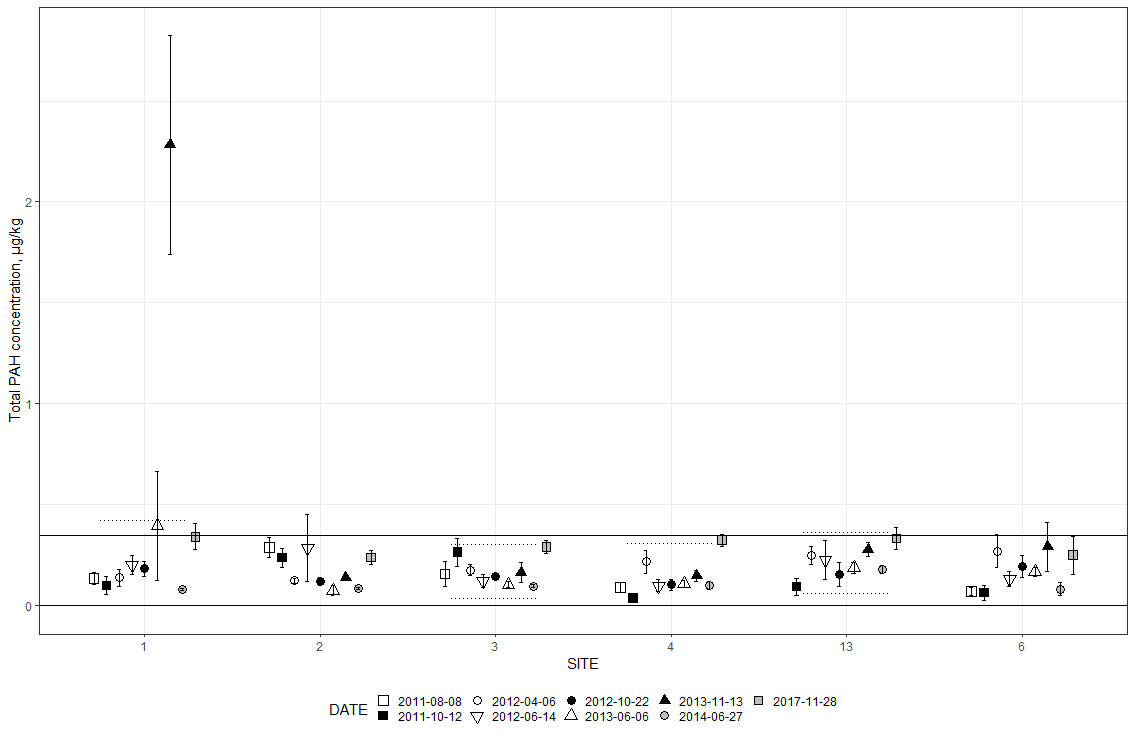


Figure S10. Mean mercury concentration (on all sampling dates) at reference sites. Error bars are ±SE. Dotted lines – ‘normal’ range for each site; Short dashed lines – ‘normal’ range for Sites 2 and 3 together; Long dashed lines – ‘normal’ range for Outer Harbour sites; Solid line – normal range for all reference sites together. ‘Normal’ ranges are 2SD above and below the grand mean for each site / group of sites. An overall normal range for the Inner Harbour is not marked, as Site 1 appears distinct from Sites 2 and 3. Mercury data were not available for 2017.


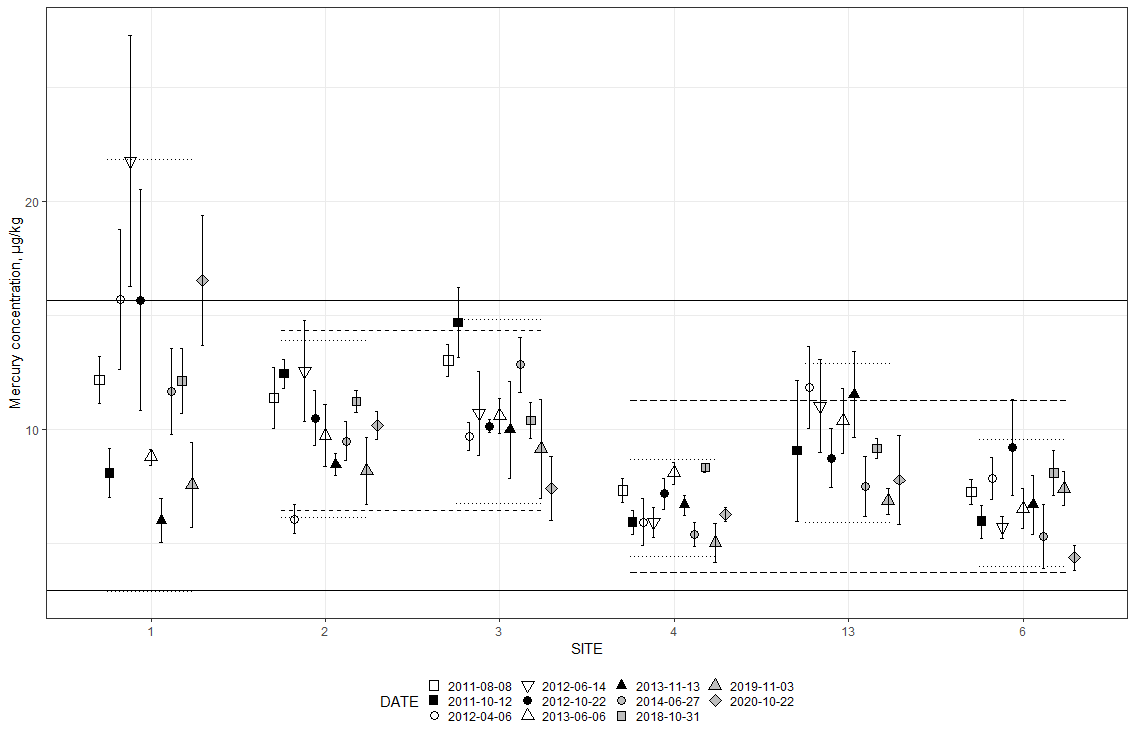


Figure S11. Mean PC1-PC3 values on all sampling dates from 2011-2019 for UNB metals data.


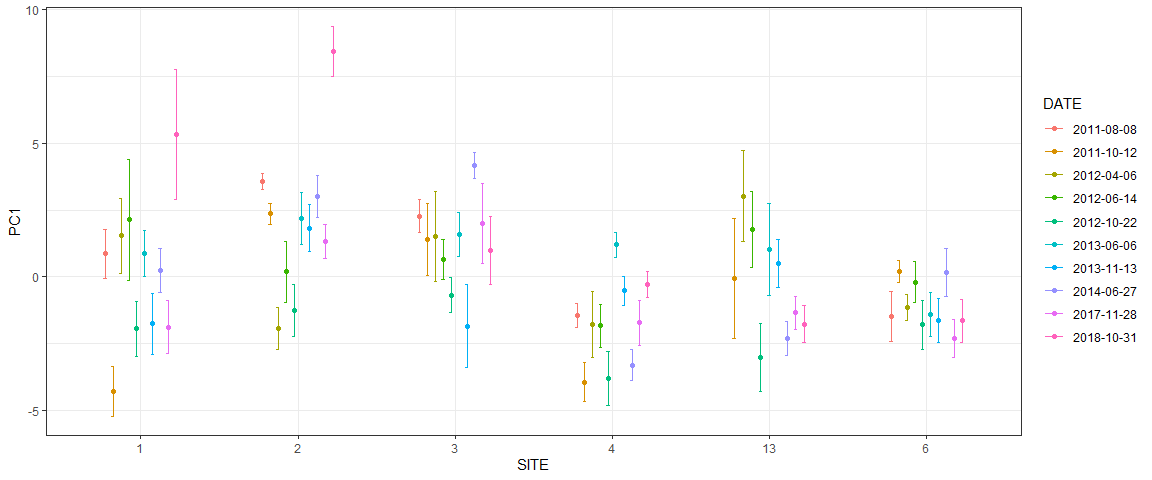


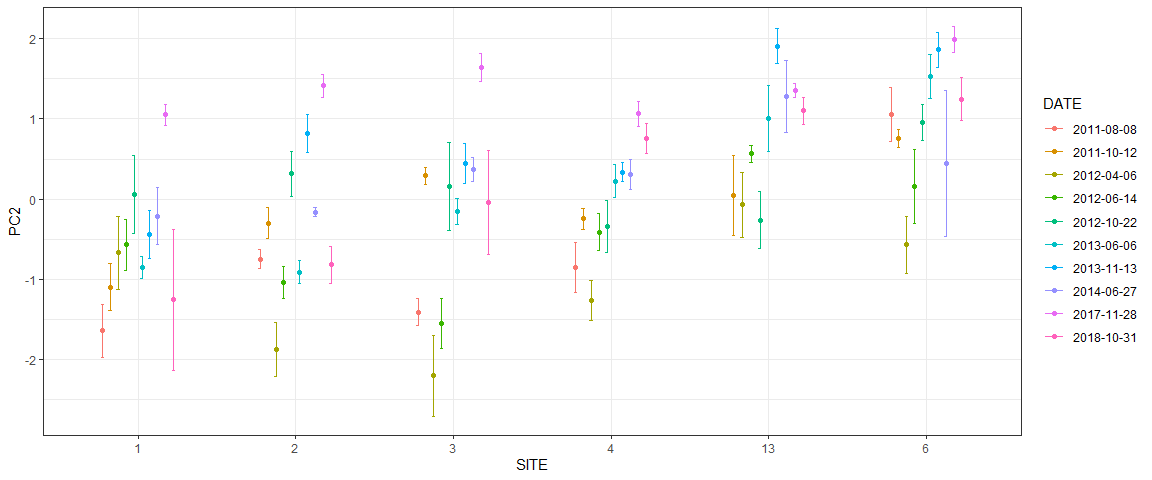


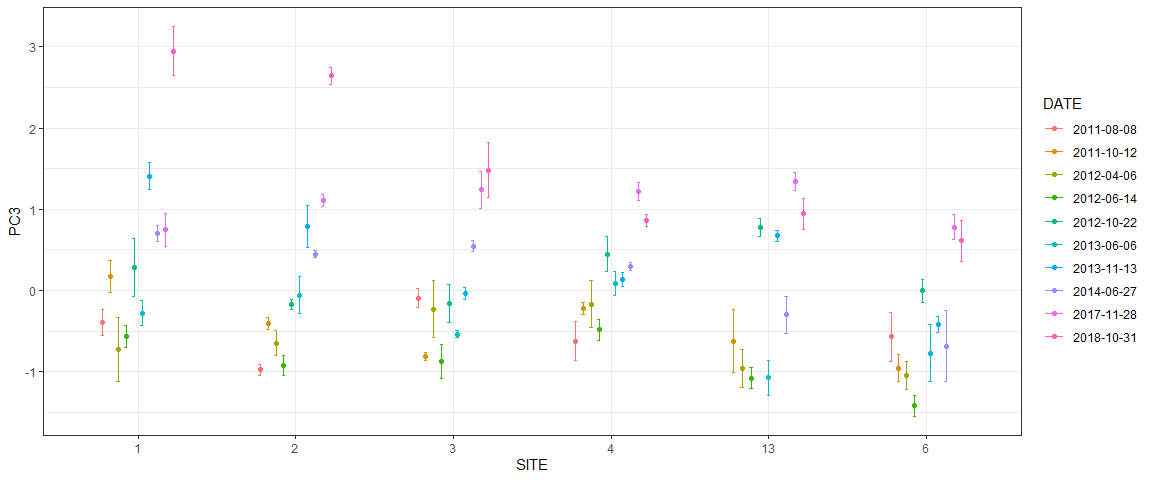


Figure S12. Average concentrations of lanthanum (representative of PC2) and lead (representative of PC3) at the reference sites, for UNB metals data (2011-2018). Replicates are mean concentrations on each sampling date (n = 10 except for Site 13, n = 9). Boxes marked with different letters are significantly different (p < 0.05) in Tukey HSD pairwise tests following significant (p < 0.05) ANOVA.


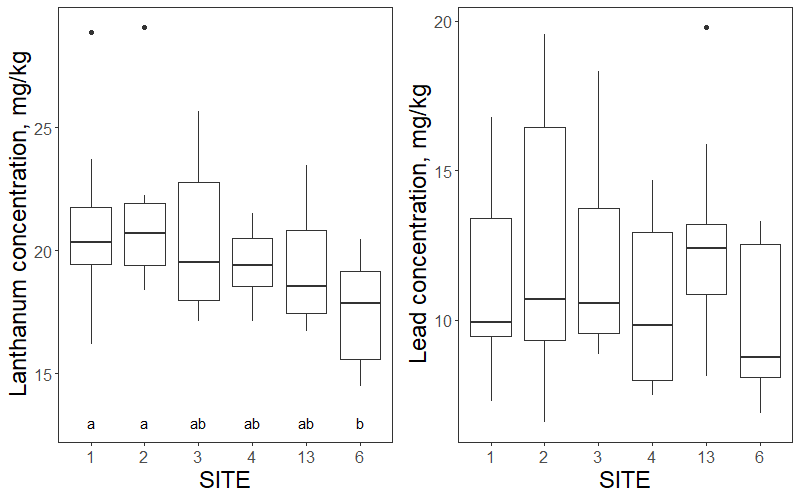


Figure S13. Average concentrations of chromium and lead at the reference sites, for RPC metals data (2018-2021). Chromium data (left panel): replicates are mean concentrations on each sampling date (n = 4). Boxes marked with different letters are significantly different (p < 0.05) in Tukey HSD pairwise tests following significant (p < 0.05) ANOVA. Lead data (right panel): mean concentration for each site on each sampling date, error bars are ± 95% confidence intervals.


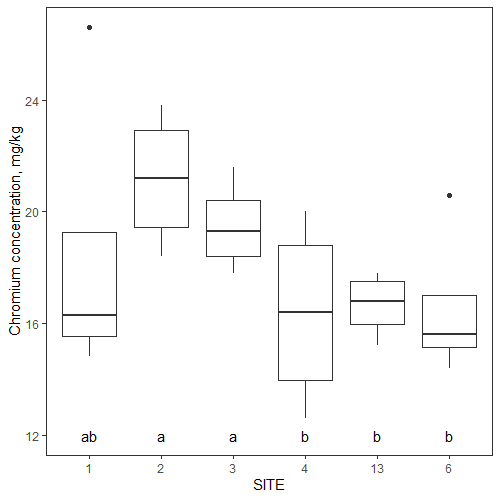

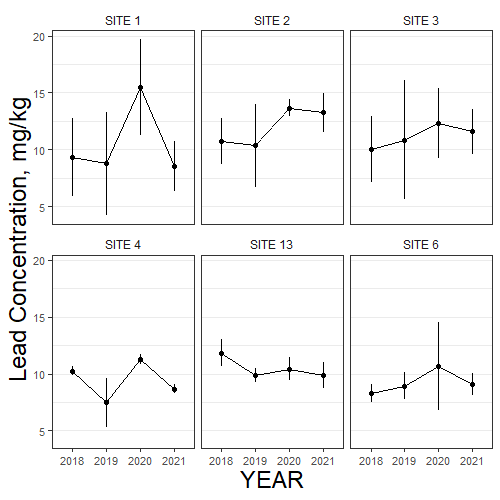

Supplement: Supplementary file 1 — Supplementary file1 (DOCX 951 KB) [file 12237_2023_1222_MOESM1_ESM.docx]
